# ROBO1 enhanced esophageal carcinoma cell radioresistance through accelerating G3BP2-mediated eIF3A degradation

**DOI:** 10.1038/s41419-025-07604-1

**Published:** 2025-04-06

**Authors:** Chunmei Zhai, Xiaorong Sun, Song Zhang, Ligang Xing

**Affiliations:** 1 Shandong Provincial Key Laboratory of Precision Medicine, Shandong Cancer Hospital and Institute, Shandong First Medical University, Jinan, Shandong China; 2 Department of Nuclear Medicine, Shandong Cancer Hospital and Institute, Shandong First Medical University and Shandong Academy of Medical Sciences, Jinan, China; 3 Shandong Provincial Key Laboratory of Precision Medicine, Shandong Cancer Hospital and Institute, Shandong First Medical University Affiliated Tumor Hospital, Jinan, Shandong China; 4 Department of Radiation Oncology, Shandong Cancer Hospital and Institute; Shandong First Medical University and Shandong Academy of Medical Sciences, Jinan, Shandong China

**Keywords:** Cancer of unknown primary, Radiotherapy

## Abstract

Radiotherapy, as a vital means of esophageal cancer treatment, has benefited countless cancer patients, but owing to the occurrence of radio-resistance, its therapeutic efficiency has been dramatically mitigated. Discovering key biomarkers governing radio-tolerance in esophageal cancer and revealing their inherent molecular mechanisms will be of great significance for clinical cancer treatment. Here, we have found roundabout guidance receptor 1 (ROBO1) was significantly upregulated in esophageal cancerous tissues and showed enhanced expression with the development of cancer staging. Cellular experiments demonstrated ROBO1 directly interacted with eukaryotic translation initiation factor 3A (eIF3A) and accelerated its degradation in esophageal cancer cells after irradiation treatment. Mass spectrum analysis further revealed that in response to irradiation, ROBO1, eIF3A and G3BP2 (Ras GTPase-activating protein-binding protein 2) formed a hetero-complex and triggered lysosomes-mediated protein degradation. Knocking down of G3BP2 abrogated the influence of ROBO1 on eIF3A instability. Besides, ROBO1-mediated eIF3A degradation interrupted P53 translation process which in turn provoked downstream mTOR signaling and increased DNA repair associated genes expressions, resulting in radio-resistance enhancement in cancer cells. In conclusion, our findings revealed a novel role of eIF3A in modulating P53/mTOR signaling activity and provided a drug candidate (ROBO1) for overcoming radio-resistance in esophageal cancer.

## Introduction

As one of the most aggressive malignant tumors in the world [1], esophageal cancer is always diagnosed at the advanced stage, dramatically reducing the patients’ chances of receiving effective treatment. Among all kinds of esophagus cancer types, squamous cell carcinoma is the most common subtype in China [2]. The guidelines of cancer diagnosis and treatment have clearly stated surgery and radiotherapy are the most important means for esophageal cancer treatment [3]. However, some patients showed increased resistance to radiotherapy, which greatly dampened its treatment efficacy [4], and will inevitably lead to the recurrence and poor prognosis of the cancer [5]. What causes the occurrence of radiation resistance? DNA double-strand breaks triggered by ionizing radiation are the leading cause of cell death. To avoid radiation-induced cell death, cancer cells can recruit DNA repair and cell cycle checkpoints machinery to enhance radio-resistance [6]. Recently, the crucial homologous recombination protein, MRE11 is demonstrated to be lactylated at K673 by the CBP acetyltransferase in response to radiation-induced DNA damage, and its lactylation promotes its binding to DNA, facilitates DNA end resection [7]. What’s more, AEG upregulation confers radioresistance to esophageal squamous cell carcinoma by slowing PARP1-mediated homologous recombination and DNA repair associated proteins degradation [8]. Up to date, some progress have been made, but elucidating the molecular mechanisms underlying ESCC radiosensitization and identifying potential targets to overcome radioresistance are still challenges for cancer researchers.

Ring-binding receptor 1 (ROBO1) was first identified as a conserved transmembrane receptor protein that played a key role in neurodevelopment and signal transduction [9]. Later studies have confirmed that ROBO1 is wildly expressed in a variety of non-neural tissues, such as esophagus, breast, lung, digestive systems, etc [10]. SLIT2 was reported as the major ligand for ROBO1 and their interaction stimulated downstream signal cascades in cancer cells to exert contrasting function in different cancer types [11]. In breast cancer cells, PRRG4 via NEDD4-mediated ubiquitination to downregulate ROBO1, resulting in the activation of Src and FAK and enhanced breast cancer metastasis [12]. But in small cell lung cancer, SLIT2 and ROBO1 showed opposing expression pattern in cancerous and normal tissues, molecular analysis showed that ROBO1 knockout or SLIT2 overexpression suppressed the transforming growth factor beta 1/β-catenin signaling pathway in tumor cells [13]. Even in the same cancer type, the outcomes induced by the coadoption of SLIT2/ROBO1 are different. YY1 inhibited pancreatic cancer growth through upregulating ROBO1 transcription [14], but the existence of ROBO1 also contributed to cancer cell liver metastatic dissemination [15]. Endothelial-derived SLIT2 protein and its receptor ROBO1 promoted the migration of lung cancer cells towards endothelial cells and invasion, but deletion of tumoral SLIT2 enhanced metastatic progression [16]. In summary, all of this data indicated the receptor independent characteristics of ROBO1.

However, in this study, we have found that in response to irradiation stress, ROBO1 directly interact with eIF3A proteins and accelerated its degradation through orchestrating G3BP2-mediated lysosomes degradation pathway in ESCC cells. Reduced eIF3A was unable to maintain P53 translation in ESCC, relieving mTOR signaling activity and amplifying DNA repair associated genes expressions. All of these results revealed the onco-promoting role of ROBO1 in strengthening ESCC radioresistance and paved a new way to improve the anti-tumor efficacy of radiotherapy in ESCC through targeting eIF3A-mediated P53 signaling.

## Materials and methods

### Cell culture

Human esophageal carcinoma cell lines KYSE450 and TE1 (HyCyte, Suzhou, China) were incubated in RPMI Medium 1640 basic (Gibco, USA) containing 10% fetal bovine serum (OriCell, China) and 1% Penicillin-Streptomycin Solution (Biosharp, China). HEK293T cells kindly provided by Shanghai Cell Bank, Chinese Academy of Sciences, were cultured in Dulbecco’s modified Eagle’s medium (Gibco, USA) supplemented with 10% fetal bovine serum. All cells were incubated at 37 °C in a humidified atmosphere with 5% CO2.

### Cell transfection

The plasmids of pMD2G, psPAX, shRNA (Vigene Biosciences, Shandong, China) were co-transfected into HEK293T with Lipofectamine 3000 transfection kit (Invitrogen, USA) according to the manufacturer’s instructions. At 48 h after transfection, the cell culture supernatant was collected, concentrated using a 100 kDa filter (Millipore, USA) and filtered through a 0.22 µm membrane (BIOFIL, Guangzhou, China). Next, viral solution was added into the esophageal cancer cells culture medium and continued to cultivate cells for 24 h. Subsequently, the cells successfully expressing shRNAs were screened with Puromycin treatment (Solarbio, Beijing, China) at 5 μg/ml for one week. The protocol for siRNA transfection (GenePharma, Shanghai, China) is same to the plasmids using Lipofectamine 3000 transfection kit (Invitrogen Carlsbad, CA, USA). All of the sequences used for shRNAs and siRNAs are provided in the Supplements.

### Real-time quantitative PCR assays

The total RNA was extracted with an RNA-easy isolation reagent (Vazyme, Nanjing, China) as described in the protocol, and the complementary first-strand DNAs were synthesized with HiScript III RT SuperMix kit (Vazyme, Nanjing, China) following the manufacturer’s instructions. Polymerase Chain Reaction (PCR) for each gene was completed using ChamQ Universal SYBR qPCR Master Mix (Vazyme, Nanjing, China) containing specific primers. Target genes relative expression levels were calculated using ΔΔCt method and normalized by GAPDH (Glyceraldehyde-3-phosphate dehydrogenase).

### Western blot assays

After sonication and centrifugation, protein samples dissolved in RIPA Lysis buffer (Bioss, Beijing, China) were quantified with BCA Protein Quantification Kit (Beyotime, Shanghai, China). Protein lysates were separated in 4–20% density gradient gel (ACE Biotechnology, Nanjing, China) and electro-transferred to PVDF membranes (Millipore, USA). After blocking in TBST solution containing 5% skimmed milk for 2 h, the membrane were subjected to primary antibody incubation at 4 °C overnight. Next, the secondary antibodies (Seracare, USA) was added and incubated at room temperature for 2 h. Finally, the bands for target proteins were detected using ECL chromogenic solution (Yeasen Biotech, Shanghai, China).

### Cell counting Kit-8 assays

About 2000 cells were seeded into a well of 96-well plate (100 μL/well) and the density of the cells were measured at the indicated time. Briefly, 10 μL of Enhanced Cell Counting Kit-8 regent (Bioss, Beijing, China) were added into medium and incubated for 1.5 h in the darkness. Light Absorbance for each well was measured at 450 nm.

### Colony formation assays

About 2000 cells were seeded into a well of a six-well plate. At 24 h after cell seeding, the cells were exposed to irradiation and continued cell culture for 7 ~ 14 days. Cell colonies were fixed for 20 min with 4% paraformaldehyde and stained with crystal violet (Bioss, Beijing, China) for 30 min. Finally, the cell colonies were photographed and counted under a microscope.

### Cell apoptosis

About 1 × 10^5^ cells were seeded into each well of a 6-well plate and irradiated at 24 h after seeding. Later, cells were collected and re-suspended in Binding buffer premixed with Annexin V-FITC (APExBIO, USA) and Propidium Iodide (APExBIO, USA). Next, the cells were incubated at room temperature in the darkness for 20 min, and then subjected to flow cytometry analysis, immediately.

### Immunoprecipitation assays

For immunoprecipitation assays, about 1 × 10^7^ cells were suspended in Cell lysis buffer (Beyotime, Shanghai, China) containing 1× PMSF (Bioss, Beijing, China) and lysed on a rotator at 4 °C for 4 h. After lysis, the cell lysate was collected by centrifuging at 15,000 × *g* for 10 min and divided into two centrifuge tubes equally. One tube was incubated with primary antibody overnight and another centrifuge tube was incubated with IgG antibody (Beyotime, Shanghai, China) as a control. Then, the target protein was pulled down with Protein A + G Agarose beads (Beyotime, Shanghai, China) from the cell lysates by incubating at 4 °C for 3 h. Beads were washed with Cell lysis buffer (Beyotime, Shanghai, China) two times, and bound proteins were eluted in SDS-loading buffer (Beyotime, Shanghai, China) and subjected to Western blot analysis.

### Immunofluorescence assays

About 1 × 10^4^ cells were seeded into a small glass-bottom round dish (Corning, USA) one day before experiment. First of all, the cells were fixed in 4% paraformaldehyde for 20 min, followed by permeabilization for 10 min with 0.3% Triton X-100 (Biosharp, China). After washing with PBS, the dishes were blocked with PBS containing 0.5% Bovine Serum Albumin (Solarbio, Beijing, China) and incubated with primary antibodies at 4 °C overnight. Subsequently, dishes were incubated with fluorescent group-conjugated secondary antibodies for 2 h in the darkness, and finally stained with DAPI (Beyotime, Shanghai, China) for 10 min. Images were visualized and recorded using a confocal laser scanning microscope (Zeiss, Germany).

### Protein degradation assays

In order to characterize the protein degradation rate of EIF3A, cycloheximide (CHX, MCE, USA) was added into the culture medium at 100 μg/ml final concentration, which resulted in the blockade of protein synthesis. Next, cell samples were collected at specific time points and the expressions of target proteins was detected using western blot.

### Lysosomes extraction and purification

Lysosomes were purified from cancer cells using a lysosomes isolation kit (Solarbio, China, Cat: EX2670) according to Lin’s method [17] with minor modifications. Briefly, about 2 × 10^7^ cells were washed with PBS and collected by gentle scraping. Then, the cell sediment was re-suspended with 4 ml of lysosomal extraction reagent A (Solarbio, Beijing, China) and homogenized with a Dounce’s homogenizer for about 40 strokes. Following homogenization, the cell extracts were transferred to new 1.5 ml tubes and centrifuged at 1000 × *g* for 10 min at 4 °C twice to ensure cell debris and nucleus clear. Then, the rough mitochondria and lysosomes fraction (MLF) was sedimented by centrifugation at 13,000 × *g* for 10 min at 4 °C and re-suspended in 600 μl lysosomal extraction reagent A containing protease inhibitor cocktail. The MLF was divided into two quits and one was incubated with IgG, another was incubated with 10 μl LAMP2 antibodies at 4 °C for two hours. Following rotation, equal amounts of PBS-washed BeyoMag™ Protein A + G Magnet beads (Beyotime, Shanghai, China) was added into each group and continued the incubation at 4 °C for another 2 h. Finally, the lysosomes are collected and washed by physical magnetic enrichment and subjected to Western blotting.

### Animals

Xenografted mice models were established through subcutaneously injecting cancer cells into BALB/c nude mice. Briefly, cancer cell suspensions were mixed with the matrix gel (Corning, USA) at the ratio of 4:1, and then subcutaneously injected into the right dorsal side of 4-week-old female nude mice at a dose of 5 × 10^6^/mice. When tumor volume reaches 200 mm^3^, mice were exposed to radiation treatment at a dose of 2 Gy every five days. If tumors were larger than 1 cm in length, mice were sacrificed to collect tumors for subsequent experiments.

### Ethics approval and consent to participate’ statement

All clinical tissue specimens were collected from Shandong Cancer Hospital. This study was approved by the Ethics Committee of Shandong Cancer Hospital and Institute (approval number: SDTHEC2023009012). The authors state that informed consent was obtained from all participants. All animal experiments were conducted in accordance with guidelines of a protocol approved by the Animal Care and Use Committee of Shandong Cancer Hospital.

### Statistics

The Graph Pad Prism 8 software was used for statistical analysis. The differences between two independent groups were assessed with the Student’s *t* test analysis. For the comparison of samples above two groups, which was analyzed by One-way ANOVA. At *p* < 0.05, the results were calculated as being statistically meaningful.

## Results

### High expression of ROBO1 is associated with poor prognosis in ESCC

**Fig. 1:**
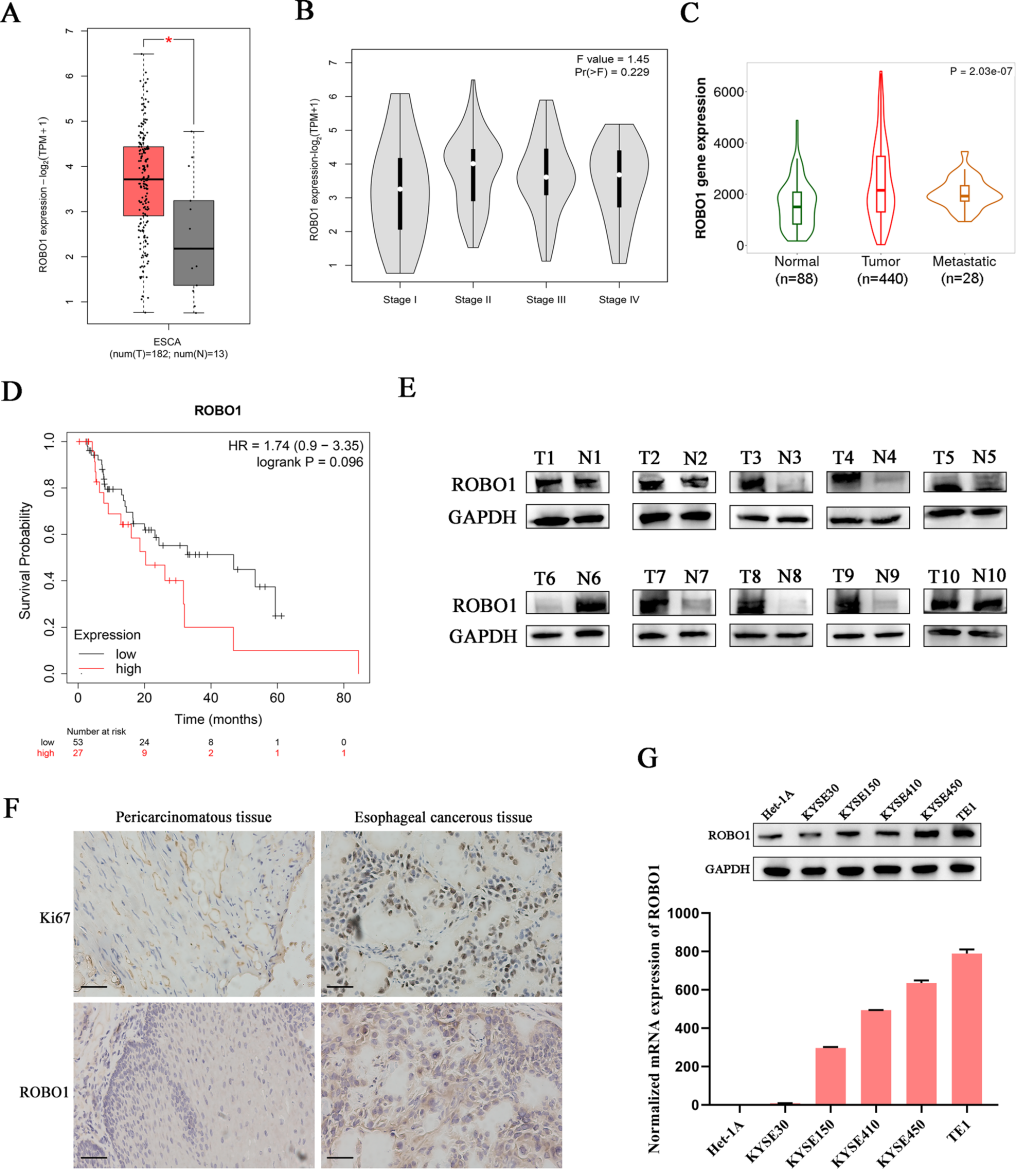
High expression of ROBO1 is associated with poor prognosis in ESCC. **A** The mRNA expression level of ROBO1 in esophageal cancerous tissues (n = 182) and normal tissues (n = 13) was analyzed using GEPIA database. *p < 0.05. **B** The expression of ROBO1 in patients with ESCC at different stages was investigated. **C** The relationship between ROBO1 expression and ESCC metastasis were evaluated by online TNMplot database. **D** Kaplan–Meier curves showed the correlation between ROBO1 expression and overall ESCC patient survival by Kaplan–Meier Plotter. **E** ROBO1 protein expression in esophageal cancerous tissues and pericarcinomatous tissues was detected by western blot. **F** Representative images of ROBO1 and Ki67 staining in esophageal cancerous and pericarcinomatous tissues were presented. Scale bars, 50 μm. **G** Different ESCC cell lines were subjected to ROBO1 detection by western blot (upper) and qRT-PCR (bottom).

In order to explore the function of ROBO1 in ESCC, the expression level of ROBO1 in patient-derived samples was analyzed by referring to some online databases, including GEPIA (http://gepia.cancer-pku.cn/), TNMplot (https://tnmplot.com) and Kaplan–Meier Plotter (http://kmplot.com). The results showed that ROBO1 was significantly up-regulated in ESCC cancerous tissues, especially in metastasis, compared with the normal tissues (Fig. 1A, C). With the progression of ESCC, ROBO1 expression also showed an obvious upregulation (Fig. 1B). Besides, ESCC patients with higher ROBO1 expression always exhibited shorter survival time when compared with ROBO1-lower group (Fig. 1D). In accordance with the online database analysis, the majority of clinical ESCC cancerous tissues we collected also presented higher ROBO1 expression relative to the paired normal tissues (Fig. 1E, F). All of this data indicated ROBO1 may play an onco-promoting role in ESCC progression. To further investigate the function of ROBO1 in ESCC, the level of ROBO1 in several cancer cell lines was detected. KYSE450 and TE1 showed the highest ROBO1 expression among all selected cell lines (Fig. 1G), which indicated these two cell lines are more suitable for the next research work.

### Silencing ROBO1 expression improved ESCC cells radio-sensitivity

**Fig. 2:**
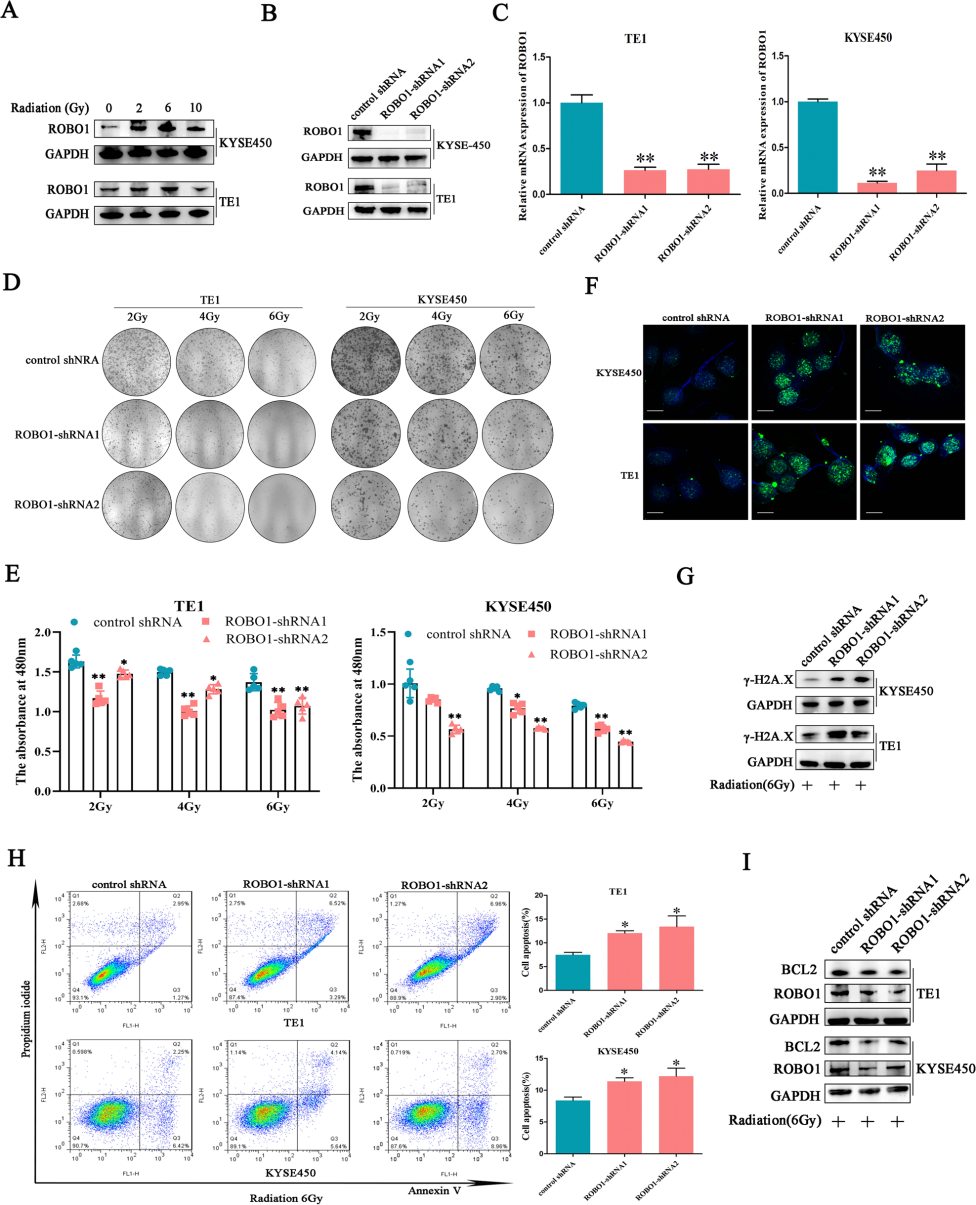
Silencing ROBO1 expression improved ESCC cells radio-sensitivity. **A** The influence of radiation on ROBO1 expression detected by Western blot. **B**, **C** The knockdown efficiency of ROBO1 in ESCC cells was determined by a western blot and RT-qPCR assays. **D**, **E** ESCC cell proliferation status was evaluated by clone formation assays and CCK-8 assays after radiation exposure. **F** Representative immunofluorescence images of γ-H2A.X distribution in cells with or without ROBO1 depletion under radiation condition. **G** The influence of ROBO1 knockdown on γ-H2A.X expression upon radiation treatment. **H** Impact of ROBO1 silencing on cell apoptosis detected by flow cytometry analysis. **I** The expression of apoptosis-associated protein was detected in ROBO1-knockdown cells by Western blot.

To elucidate the relationship between ROBO1 and ESCC malignancy, the function of ROBO1 in mediating cancer cell radio-resistance was explored. Western blot results showed that in response to irradiation, ROBO1 level was dose-dependently increased, indicating a potential role of ROBO1 in regulating ESCC radio-sensitivity (Fig. 2A). Subsequently, ESCC cell lines with stably decreased ROBO1 were established and the shRNA knockdown efficiency was validated at mRNA level and protein level, respectively (Fig. 2B, C). As expected, when ROBO1 was knocked down, the proliferation rate and colony formation capacity of ESCC cells was attenuated under radiation condition (Fig. 2D, E). As the recruiter of DNA damage repair machinery, γ-H2A.X’s amount and nucleic distribution pattern were usually used to characterize the status of genome. In our results, compared with control group, ROBO1 depletion induced γ-H2A.X higher expression and more focal particles formation in nucleus under irradiation treatment at same time point (Fig. 2F, G), which suggested ROBO1 knockdown aggravated irradiation-induced DNA damage. Meanwhile, flow cytometry analysis uncovered stronger cell apoptosis occurred in ROBO1-silenced cells following irradiation exposure relative to the control cells (Fig. 2H). In accordance with the results of flow cytometry, BCL2, an apoptosis inhibitor, showed significantly down-regulation after ROBO1 silence (Fig. 2I). All of these findings indicated that ROBO1 played a vital role in driving ESCC malignancy through enhancing cancer cell radio-resistance.

### ROBO1 accelerated eIF3A degradation in ESCC cells

**Fig. 3:**
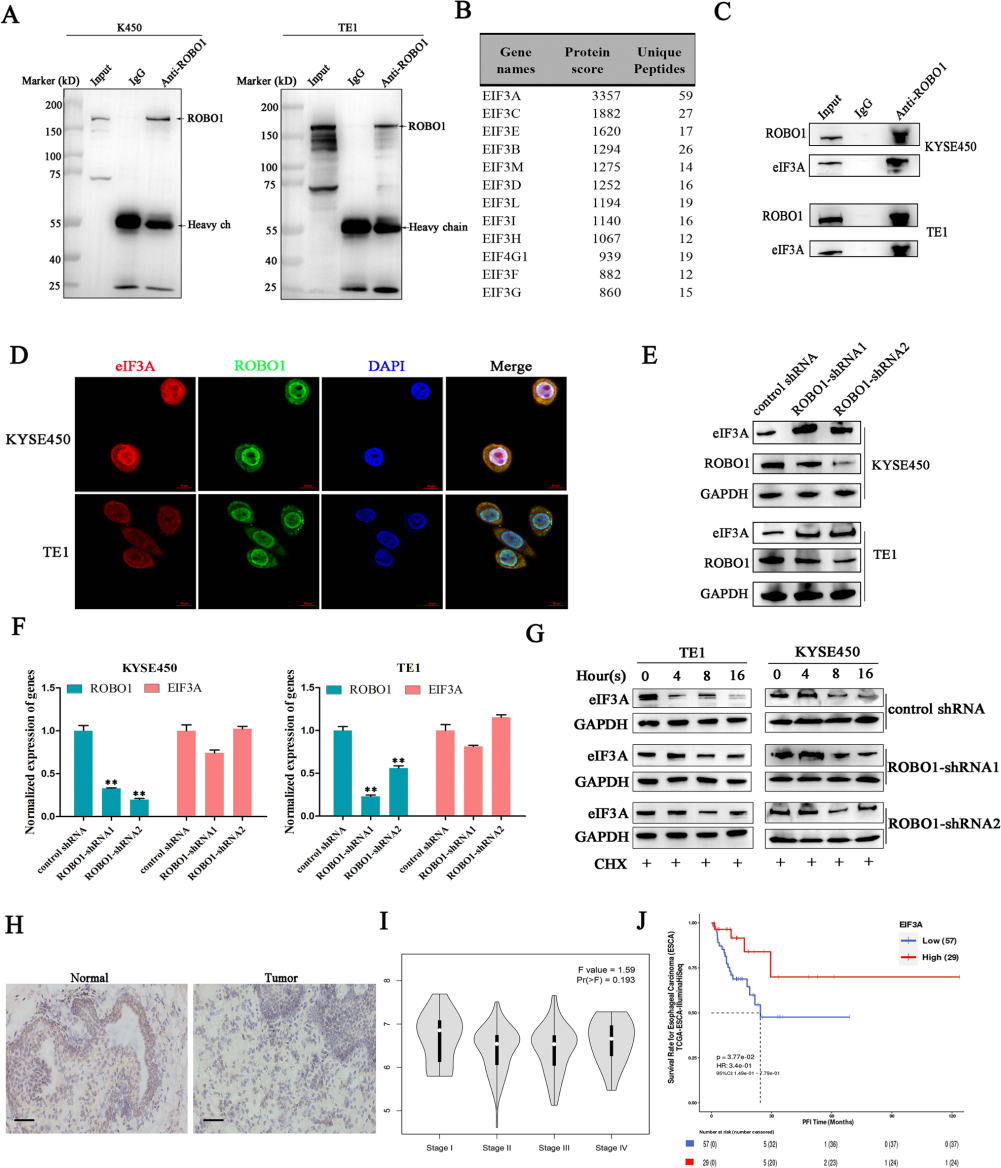
ROBO1 accelerated eIF3A degradation in ESCC cells. **A** ROBO1 was specifically immunoprecipitated with antibodies in ESCC cells. **B** LC-MS analysis. **C** Co-immunoprecipitation assays were performed to demonstrate the interaction of ROBO1 with eIF3A and G3BP2. **D** Immunofluorescent staining displayed the co-localization of ROBO1 and eIF3A in ESCC cells. **E**, **F** The influence of ROBO1 knockdown on eIF3A expression was detected at protein level and mRNA level. **p < 0.01. **G** The degradation rate of eIF3A was monitored in ESCC cells with or without ROBO1 depletion. **H** Immunohistochemical staining of eIF3A in esophagus cancerous tissues and para tissues. Scales, 50 μm. **I** The expression levels of eIF3A in ESCC patients at different stages. **J** Overall survival rate of ESCC patients with different eIF3A expressions were analyzed using a online database (https://smuonco.shinyapps.io/PanCanSurvPlot/).

To gain insight into the molecular mechanism by which ROBO1 mediated ECSS cell radioresistance, immunoprecipitation coupled with mass spectrometry analysis were employed to profile proteins interacted with ROBO1 (Fig. 3A). Among all identified proteins, the family of eukaryotic translation initiation proteins and stress granules associated proteins were found to be enriched in anti-ROBO1 sediments (Fig. 3B). To further validate this endogenous interaction, we incubated ESCC cell lysates with anti-ROBO1 antibodies and found eIF3A and G3BP2 were co-precipitated with ROBO1 in both cell lines (Fig. 3C). Besides, immunofluorescent staining demonstrated the colocalization between ROBO1 and eIF3A in ESCC cancer cells (Fig. 3D). All of this results demonstrated the interaction between ROBO1 and eIF3A was genuinely existed in ESCC cells. Intriguingly, we found ROBO1 knockdown significantly diminished the expression of eIF3A at protein level, but with no effect on its mRNA expression (Fig. 3E, F). Therefore, translation inhibitor cycloheximide (CHX) was used to block cellular de novo peptides synthesis and found silencing ROBO1 significantly decreased the degradation rate of eIF3A in ESCC cancer cells (Fig. 3G). In conclusion, ROBO1 directly interacted with eIF3A to accelerate its degradation in ESCC cells. To reveal the connection between of eIF3A expression and esophagus cancer progression, the expression level of eIF3A was investigated by immunohistochemical method, and we have found the level of eIF3A in esophagus cancerous tissues was decreased relative to the paired para-carcinoma tissues (Fig. 3H). Besides, TCGA database analysis showed eIF3A expression in ESCC decreased with the development of cancer stages, lower eIF3A expression always associated with dismal survival status for ESCC patients (Fig. 3I, J), which further indicated eIF3A functioned a tumor suppressing role in ESCC confronted with ROBO1-mediated malignancy.

### ROBO1 elicited lysosomal eIF3A degradation through interacting with G3BP2

**Fig. 4:**
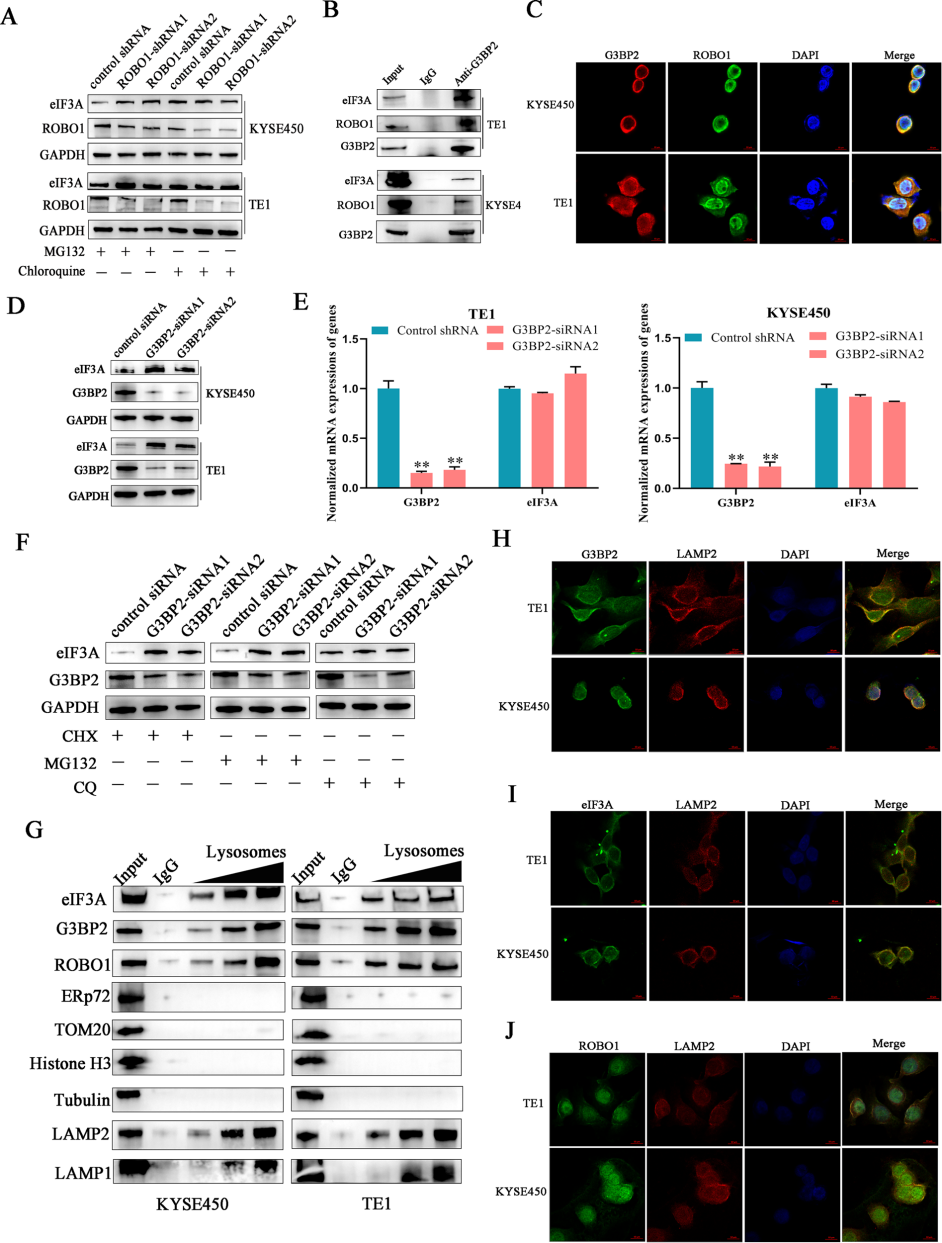
ROBO1 elicited lysosomal eIF3A degradation through interacting with G3BP2. **A** ROBO1 regulated eIF3A expression relying on lysosomes. MG132, a protease inhibitor. Chloroquine, an autophagy lysosome inhibitor. **B** G3BP2 interacted with ROBO1 and eIF3A in ESCC cells. **C** Immunofluorescence staining showed the colocalization of ROBO1 (green) and G3BP2 (red) in ESCC cells. Scale bars, 10 μm. **D**, **E** G3BP2 knockdown increased eIF3A expression at protein level, but not at mRNA level. **p < 0.01. **F** G3BP2 promoted eIF3A degradation relying on lysosomes activity. **G** Lysosomes were purified from cells and subjected to Western blot. ERp72, TOM20 and Histon3 were used as the markers of endoplasmic reticulum, mitochondria and nucleus, respectively. **H**–**J** Immunofluorescence assays demonstrated the co-localization of G3BP2, ROBO1, eIF3A with lysosomal marker protein LAMP2 (red) in ESCC cells. Nucleus were stained with DAPI (blue). Scale bars, 10 μm.

The finding that knocking down ROBO1 expression induced eIF3A upregulation at protein level but not mRNA level led us to hypothesize whether ROBO1 was involved in regulating eIF3A protein stability in ESCC cells. Therefore, the canonical protein degradation pathways including proteasome and lysosomes, were blocked using small molecule inhibitors, respectively. Intriguingly, chloroquine (inhibitor of lysosome), but not MG132(inhibitor of proteasome), successfully compromised the effects of ROBO1 silencing-induced eIF3A upregulation in ESCC cells, indicating that lysosomes may participate in modulating eIF3A protein stability (Fig. 4A). Then, we re-checked the mass spectrometry (MS) data of anti-ROBO1 and G3BP2 (Ras GTPase-activating protein-binding proteins 2) was screened out owning to its reported function in anchoring tuberous sclerosis complex (TSC) protein complex to lysosomes [18]. Immunoprecipitation and confocal assays demonstrated the direct interaction between G3BP2 and ROBO1 in ESSC cells which is in accordance with the results of MS (Fig. 4B, C). To explore whether G3BP2 was engaged in regulating eIF3A degradation like TSC, G3BP2 expression was suppressed using targeted siRNAs in ESCC cells. It was found that G3BP2 depletion significantly induced eIF3A upregulation at protein level, but not mRNA level (Fig. 4D, E). Due to eIF3A protein expression can be influenced by translation or degradation processes, different inhibitors targeting these processes were employed to detect their influence on eIF3A expression. Similar to the results of ROBO1, chloroquine was able to block G3BP2 silence-mediated eIF3A upregulation in cancer cells exposed to irradiation (Fig. 4F). To further interrogate whether lysosomes were implicated in orchestrating eIF3A degradation, the lysosomes were isolated from ESCC cells and subjected to Western blot. We can see from the results (Fig. 4G), ROBO1, G3BP2 and eIF3A were simultaneously presented in lysosomes fraction without endoplasmic reticulum, mitochondria and nucleus contamination. In line with the results of lysosomes fraction, immunofluorescent confocal assays demonstrated that all of G3BP2, ROBO1 and eIF3A were co-localized with lysosomes marker (LAMP2) in irradiation-treated ESCC cells (Fig. 4H, I). Collectively, all of mentioned results indicated that ROBO1 modulated eIF3A stability in ESCC cells through interacting with G3BP2 to trigger lysosomal degradation pathway.

### G3BP2 knockdown impaired ESCC tolerance to irradiation

**Fig. 5:**
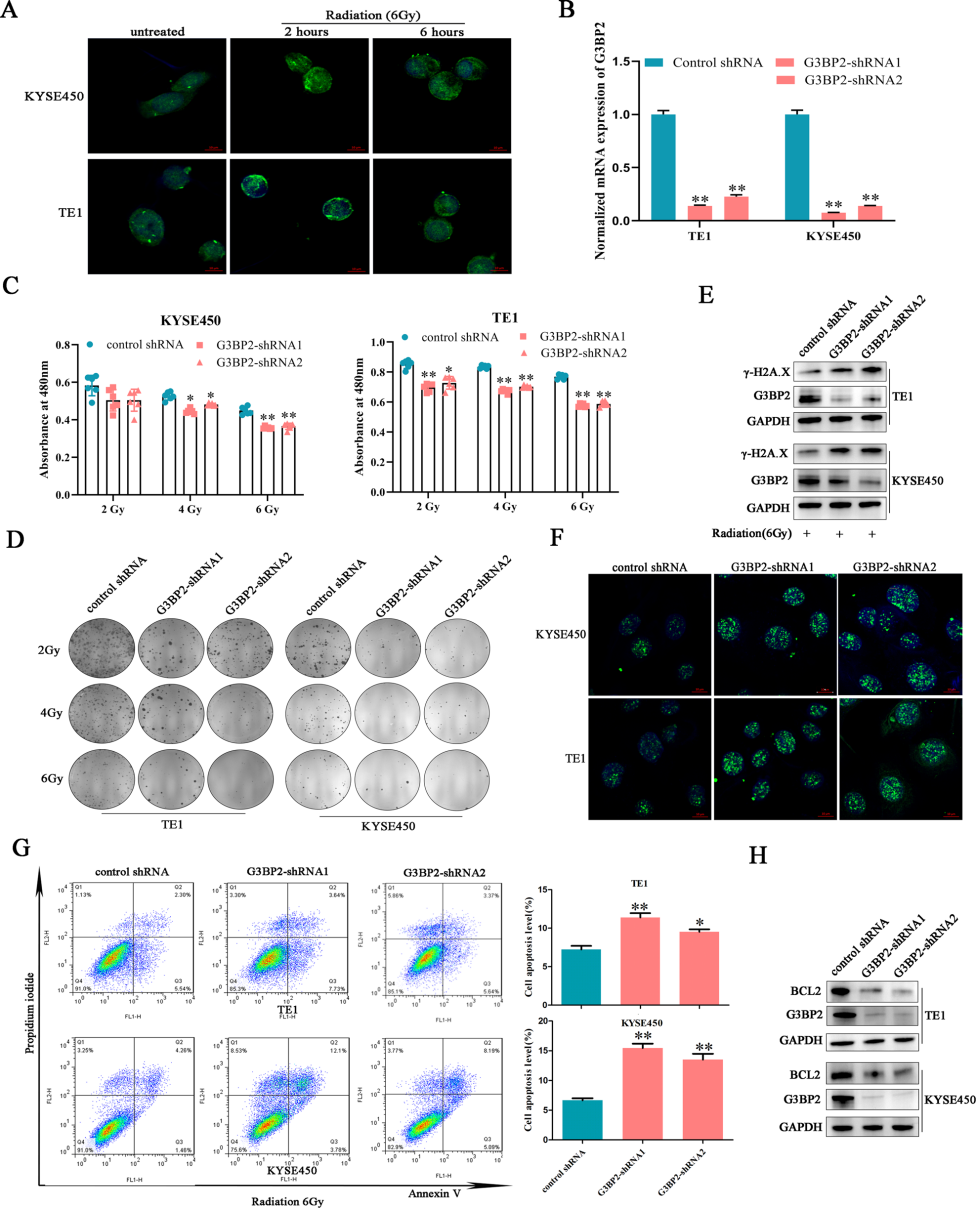
G3BP2 knockdown impaired ESCC tolerance to irradiation. **A** Irradiation triggered more G3BP2 (green) particles formation. Nucleus were stained with DAPI (blue). **B** G3BP2-silenced cells were constructed using shRNAs. **p < 0.01. **C** The survival status of ESCC cells were monitored after experiencing different doses of radiation. **D** The colony formation ability of ESCC cells was detected. **E**, **F** The influence of the G3BP2 inhibition on γ-H2A.X expression was detected by Western blot and immunofluoresent staining at 2 h after irradiation. The impact of G3BP2 silencing on cell apoptosis was detected by flow cytometry (**G**) and Western blot (**H**).

G3BP2 was reported to participate in organizing stress granules, a kind of molecular condensates that form in response to various stresses. Whether G3BP2 functioned in orchestrating ESCC radio-resistance? Immunofluorescence experiments were performed and found that G3BP2 formed more particles at 2 hours after radiation exposure and declined with time, suggesting the potential of G3BP2 as a irradiation responsor (Fig. 5A). Then, cell lines with stable decreased G3BP2 was established using lentivirus-mediated shRNA transfection (Fig. 5B). Upon irradiation treatment at different doses, ESCC cell with decreased G3BP2 presented obviously attenuated proliferation ability and clonogenicity compared with control cells (Fig. 5C, D). To explore whether G3BP2 was implicated in regulating irradiation-induced DNA damage repair process, the expression of γ-H2A.X was detected using Western blot and immunofluorescent staining. Irradiation exposure induced higher γ-H2A.X expression and longer existing time in G3BP2-depleted cells when compared with the control cells (Fig. 5E, F), suggesting that silencing G3BP2 impaired cancer cell DNA repair capability. What’s more, flow cytometry analysis identified more obvious cell apoptosis in G3BP2-depleted ESCC cells triggered by radiation, which is in accordance with the Western blot results of BCL2, an inhibitor of cell apoptosis (Fig. 5G, H). Taken together, high expression of G3BP2 rendered ESCC cells strong resistance to radiotherapy.

### ROBO1 stimulated mTOR signaling pathway through blocking eIF3A-mediated P53 translation

**Fig. 6:**
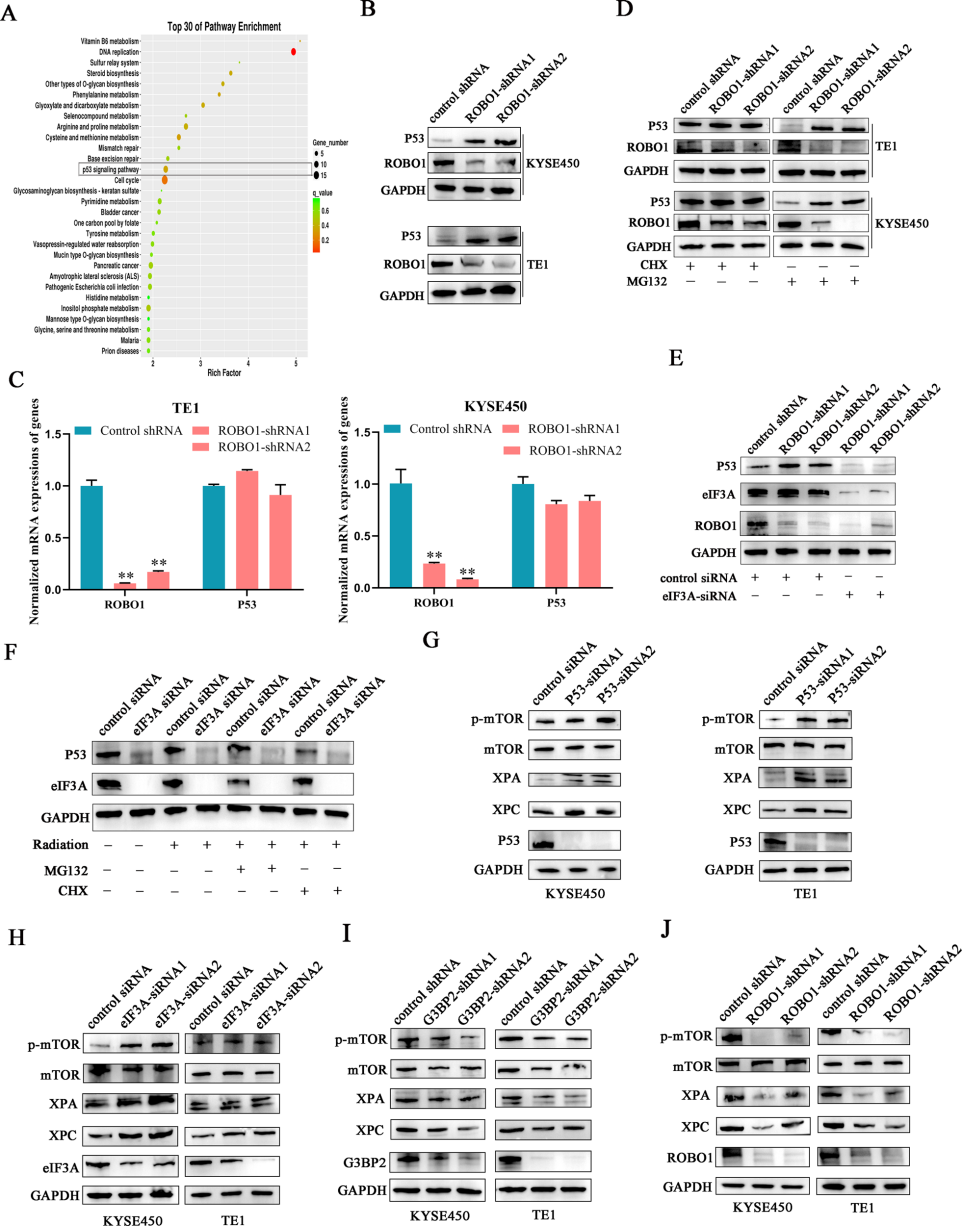
ROBO1 stimulated mTOR signaling pathway through blocking eIF3A-mediated P53 translation. **A** Pathway enrichment analysis for the influence of ROBO1 knockdown in ESCC. **B**, **C** The influence of ROBO1 silence on P53 expression was detected at protein level and mRNA level. **p < 0.01. **D** P53 protein degradation and translation was blocked in ESCC cells with or without ROBO1 depletion. MG132, a proteasome inhibtor. CHX, a translation blockade. **E** ROBO1 regulated the translation of P53 with the help of eIF3A. **F** The influence of EIF3A on regulating P53 translation and stability was explored by Western blot. **G** Silencing P53 initiated mTOR signaling cascades and induced downstream DNA nucleotide excision repair (NER) genes expressions in response to irradiation treatment. **H**–**J** The influence of G3BP2, ROBO1 or eIF3A abrogation on mTOR signaling activity was explored in ESCC cells upon irradiation exposure.

To further elucidate the mechanisms by which ROBO1 and eIF3A regulated ESCC radioresistence, cancer cells with or without decreased ROBO1 expression were subjected to RNA sequencing. Kyoto Encyclopedia of Genes and Genomes (KEGG) analysis showed that genes with altered expressions were specifically enriched in P53 signaling pathway (Fig. 6A). Furthermore, silencing ROBO1 expression in ESCC cells obviously induced P53 upregulation at protein level, but no significant difference was observed at mRNA level (Fig. 6B, C), indicating that ROBO1 post-transcriptionally regulated P53 expression. Therefore, the proteasome inhibitor (MG132) and translation blockade (CHX) were employed to interrupt the process that potentially engaged in ROBO1-mediated P53 alteration. It was shown that CHX, but not MG132, effectively compromised the upregulation of P53 induced by ROBO1 silence in ESCC cells (Fig. 6D). We hypothesized whether ROBO1 regulated P53 expression through direct molecular binding? but anti-ROBO1 immunoprecipitation assays failed to trace the direct interaction between ROBO1 and eIF3A. Surprisingly, suppressing eIF3A expression using siRNAs completely abrogated ROBO1 depletion-triggered P53 upregulation in ESCC cells (Fig. 6E), suggesting the indispensable role of eIF3A in modulating P53 elevation. After irradiation treatment, cancer cells with decreased eIF3A expression were exposed to the several inhibitors treatment, and we have found the addition of the translation inhibitor (CHX) dramatically mitigated P53 elevation initiated by eIF3A alteration (Fig. 6F), indicating that the exist of eIF3A contributed to P53 translation. Large evidence has demonstrated that P53 participated in modulating mTOR (mammalian target of rapamycin) signaling activity and suppressing its downstream genes expressions, including DNA nucleotide excision repair (NER) genes [19, 20]. Here, we have found P53 silencing significantly elevated mTOR phosphorylation and NER genes expressions in ESCC cells, which was consistent with others’ reports (Fig. 6G). To make it clear whether ROBO1 participated in regulating radiosensitivity through eIF3A/P53/mTOR pathway, the expressions of ROBO1 and eIF3A were suppressed in ESCC cells. We have found eIF3A silencing obviously stimulated mTOR signaling activity and enhanced NER gene expression (Fig. 6H), contrary to the results observed in G3BP2 or ROBO1 abrogated cells (Fig. 6I, J). All of these data demonstrated that ROBO1 tuned ESCC radiosensitivity through eIF3A/ P53/mTOR axis.

### Disrupting ROBO1/G3BP2/eIF3A axis effectively overcame the resistance of ESCC to radiation in vivo

**Fig. 7:**
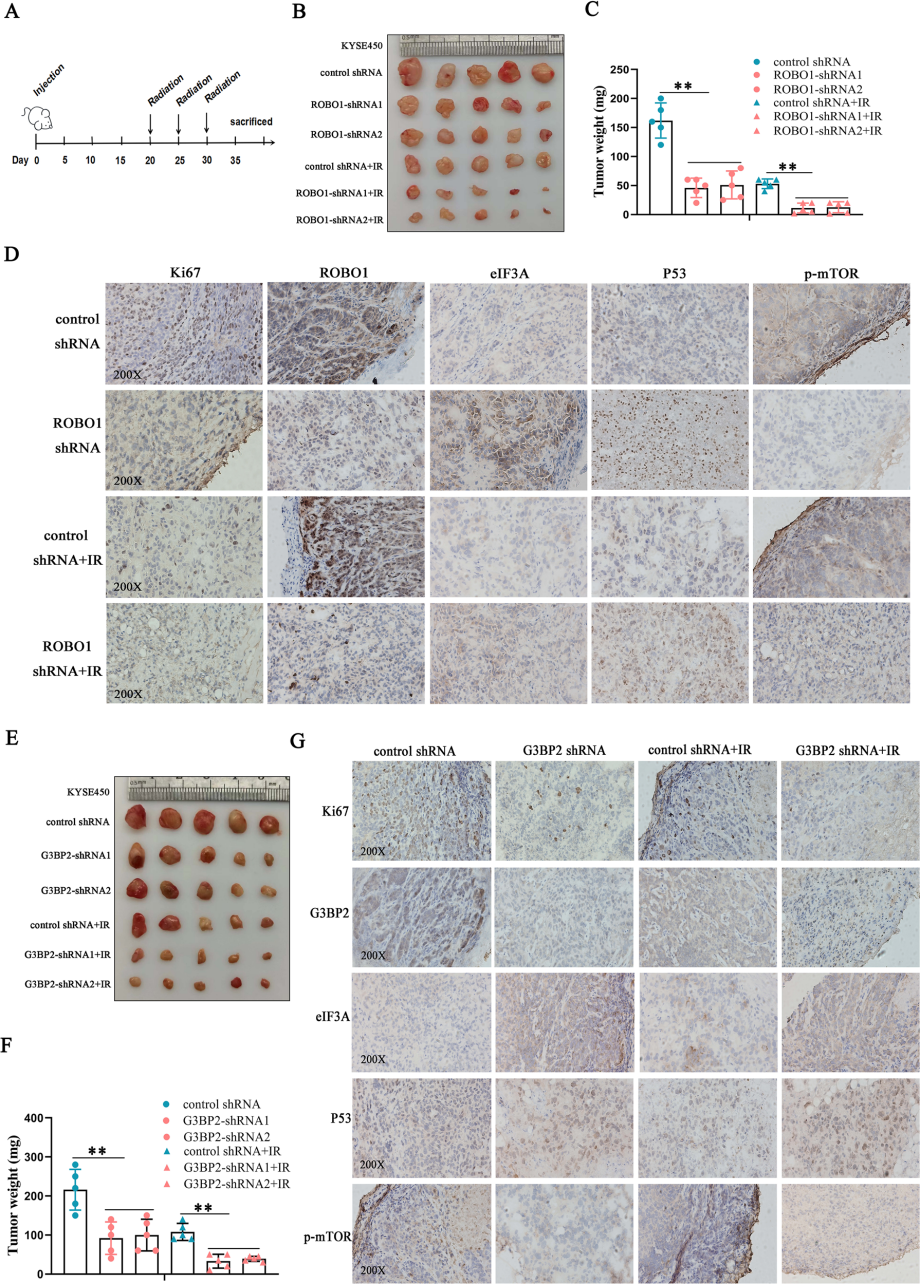
Disrupting ROBO1/G3BP2/eIF3A axis effectively overcame ESCC radio-resistance in vivo. **A** Schematic illustration of tumor inoculation and irradiation process for building mice models. **B** Representative picture of xenografted solid tumors dissected from mice exposed to irradiation (IR) or not. **C** Tumor weights analysis. **p < 0.01. **D** Representative immunohistochemical staining pictures of Ki67, ROBO1, eIF3A, P53 and phosphorylated mTOR in xenografted solid tumor tissues with or without ROBO1 deficiency. **E** Representative images of xenografted solid tumors with or without eIF3A depletion. **F** Statistical analysis of tumor weights. **p < 0.01. **G** Representative immunohistochemical staining pictures of solid tumor tissues with or without G3BP2 decrease.

To further elucidate the function of ROBO1 in regulating ESCC tumorigenesis in vivo, xenografted tumor models with or without ROBO1 knockdown were established subcutaneously. Compared with the control group, ROBO1 depletion significantly reduced tumor burdens and the tumor-suppressing effect was further amplified especially when mice were exposed to irradiation (Fig. 7B, C), suggesting that ROBO1 functioned in strengthening radio-resistance in ESCC. In addition, immunohistochemical (IHE) staining results showed that silencing ROBO1 significantly increased eIF3A and P53 expression and inhibited mTOR phosphorylation in ROBO1-deficient xenografted solid tumor tissues in relative to the control group, which is consistent with the observed cellular results (Fig. 7D). Whether G3BP2-mediated eIF3A lysosomal degradation was implicated in regulating ESCC cell growth and radiotherapy tolerance in vivo, tumor models with decreased G3BP2 were established in nude mice. Similarly, G3BP2 deficiency obviously mitigated tumor growth in mice and efficiently impaired the ability of cancer cell to tolerance radiotherapy in vivo (Fig. 7E, F). IHE staining revealed a negative correlationship between G3BP2 and eIF3A expressions in cancerous tissues (Fig. 7G). Besides, increased P53 expression and downregulated mTOR phosphorylation were presented in G3BP2-decreased tissues, further demonstrating the critical role of G3BP2 in modulating ESCC response to irradiation. Collectively, all of these results demonstrated that disrupting ROBO1 or G3BP2 mediated eIF3A protein stability will be beneficial for improving the efficiency of radiotherapy and limiting the progression ESCC.

## Discussion

Over the past decades, numerous studies have revealed the tumor-promoting role of SLIT2/ROBO1 in several cancer types, including prostate cancer, hematopoietic neoplasm and lobular breast cancer, etc [21, 22]. Traditionally, the interaction of SLIT2 and ROBO1 was able to drive carcinogenesis through directly triggering a series of downstream signaling pathways in cancer cells. In tumor environment, melanoma cells could recruit endothelial cells to construct vessels in a ROBO1-dependent manner through releasing soluble SLIT2 proteins [23], which further increased the functional complexity of ROBO1. However, the marker of gene silencing expression, hypermethylation, was uncovered within the promoter regions of ROBO1 and SLIT2 in non-small cell lung, ovarian, glioma, hepatocellular, and colorectal cancers [21, 24], which may indicating the detrimental function of SLIT2/ROBO1 for cancer progression. In our study, we have found ROBO1 was upregulated in ESCC compared with normal tissues and rendered cancer cells enhanced resistance to radiotherapy. Knocking down of ROBO1 obviously restricted ESCC cancer cell proliferation in vitro and in vivo especially when exposed to irradiation. In breast cancer cells, Slit induced redistribution of Robo1 from intracellular compartments to the plasma membrane dependent on USP33 [25], and then ROBO1 was cleaved by metalloproteinases and c-secretase and migrated into the nucleus [26], indicating that the cellular location of ROBO1 was not limited to cytoplasm membrane. Our results confirmed ROBO1 interacted with eIF3A and G3BP2 and anchored to lysosomes to regulate eIF3A stability in ESCC cytosol, but the possibility of ROBO1 function as a membrane receptor during radiotherapy should not be fully ruled out and more research works need to be performed.

Translation is a multi-step process that in charge of almost all protein-encoding genes expressions. During this process, the eukaryotic translation initiation factors are major players with at least 12 members participation in a specific spatiotemporal manner [27]. Among all of them, eIF3 is the largest and most complex factor, comprising 13 subunits designated from eIF3a to eIF3m [28]. As the largest isoform of eIF3 family, eIF3A plays a key role in translation, which has been widely reported to be implicated in tumorigenesis, cell differentiation, DNA synthesis and repair, fibrosis, etc [29]. In human non–small cell lung cancer, eIF3a promotes glucosemetabolism and cell proliferation via promoting small GTPase Rheb synthesis to orchestrate AMPK activation [30]. Depletion of eIF3a significantly reduced HIF1α protein level and cellular glycolysis ability in hepatocellular carcinoma cells through controlling internal ribosomal entry site dependent translation [31]. However, more and more evidences pointed that eIF3a is not deemed essential for the function of eIF3, and not all of eIF3a is linked with ribosomes, indicating that it may have functions beyond protein translation. It has been reported that eIF3A was able to bind with SHC and Raf-1, two components of the ERK pathway, to competitively confront EGF-induced neuron cell differentiation [32]. Besides, eIF3a was required for non-small cell lung cancer stem cell-like traits maintenance through directly regulating β-catenin and YY1 expressions at mRNA level, but not at the translational level [33]. Although the function of eIF3A in various cancers was extensively studied, but its role in ESCC and specially in orchestrating cancer cell radio-resistance is still elusive.

**Fig. 8:**
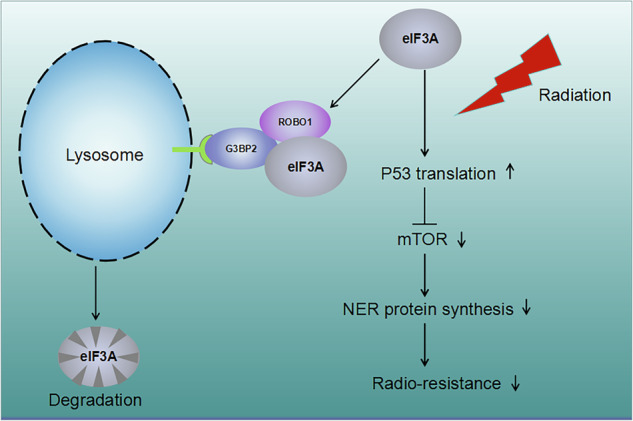
Schematic diagram deciphering the mechanisms by which ROBO1, G3BP2 and eIF3A form hetero-complex to mediate radio-resistance in ESCC cells. Mechanistically, ROBO1 interacted with G3BP2 and eIF3A to form heterocomplex and accelerated eIF3A degradation through activating lysosome signaling pathway in ESCC cells. Under irradiation exposure, free eIF3A inactivated mTOR signaling and downreglated its downstream DNA nucleotide excision repair (NER) genes expression through directly promoting P53 translation, which finally attenuated ESCC radioresistance and retarded cancer severity.

RNA sequencing revealed the p53 signaling pathway was influenced significantly by ROBO1 knocking down in ESCC cells. P53 is the single most commonly mutated gene in human tumors and has been linked to various biological processes regulations. In response to DNA damage, P53 usually activates damage-specific repair mechanisms, or in irreparable damage, activates cellular senescence, or programmed cell death to maintain genome integrity [34]. In retinoblastoma models, the mTOR activity was markedly increased in p53-deficient tumors and rapamycin treatment suppressed tumor cell growth, identifying mTOR inhibition as a critical p53 tumor suppressive function [35]. Here, we have revealed that eIF3A is required for boasting P53 translation in ESCC to trigger cell apoptosis in response to irradiation treatment. Mechanically, ROBO1 interact with eIF3A and G3BP2 to form a heterocomplex neighboring lysosomes to accelerate eIF3A degradation, resulting in P53 translation disruption. When P53-mediated mTOR signaling inhibition was relieved, downstream signaling initiated the upregulation of DNA nucleotide excision repair (NER) associated genes, including XPC and XPA, which in turn enhanced ESCC cells ability to cope with irradiation treatment (Fig. 8). Overall, the findings of this study implied that ROBO1 contributed to the enhancement of ESCC radioresistance through regulating P53/mTOR signaling and provided ROBO1 as a potential target for the treatment and prognosis of ESCC.

## Supplementary information

Original Data

supplements

## Data Availability

All the data supporting the findings of this study are available from the corresponding author on reasonable request.
