# Supplementary material for: ROBO1 enhanced esophageal carcinoma cell radioresistance through accelerating G3BP2-mediated eIF3A degradation
Source: Cell Death Dis. 2025 Apr 6;16(1):256. doi: 10.1038/s41419-025-07604-1 (PMC11972380; doi:10.1038/s41419-025-07604-1)

Fig 1E

GAPDH

T1 T2 T3 T4

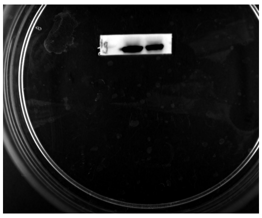 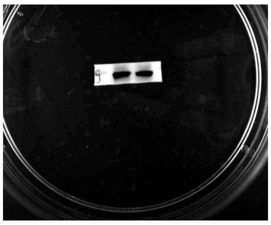 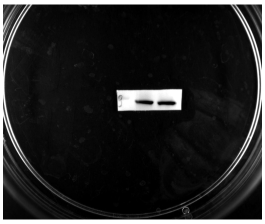 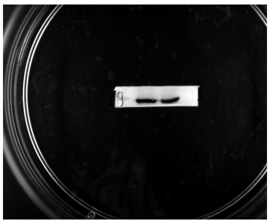

T5 T6 T7 T8

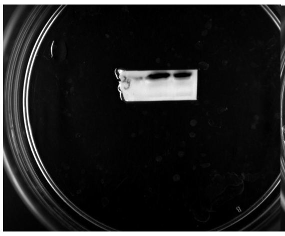 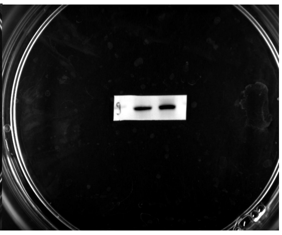 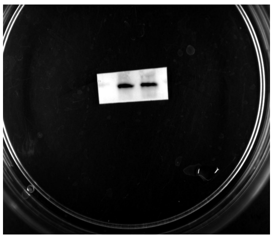 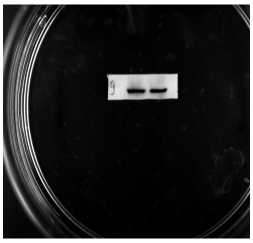

T9 T10

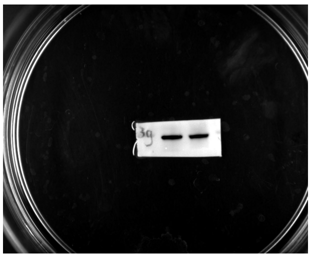 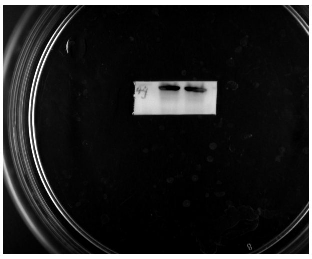

ROBO1

T1 T2 T3 T4

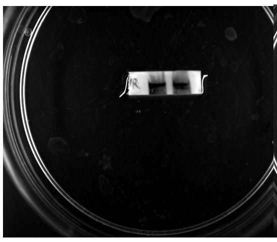 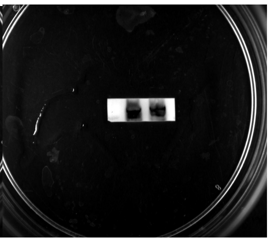 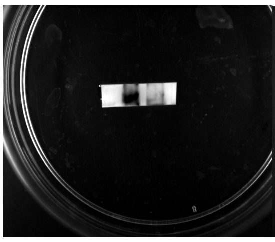 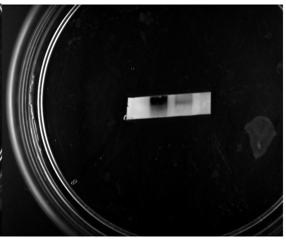

T5 T6 T7 T8

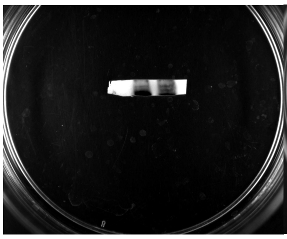 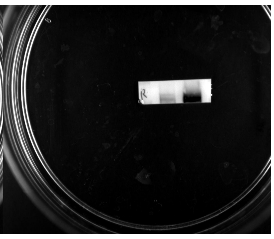 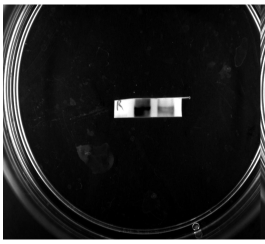 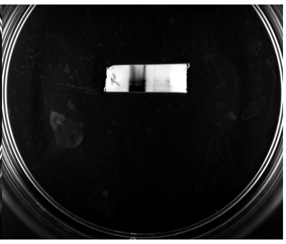

T9 T10

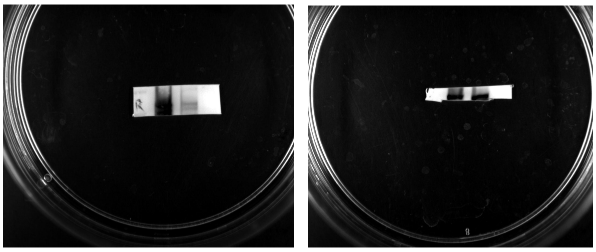

Fig 1G

ROBO1 GAPDH

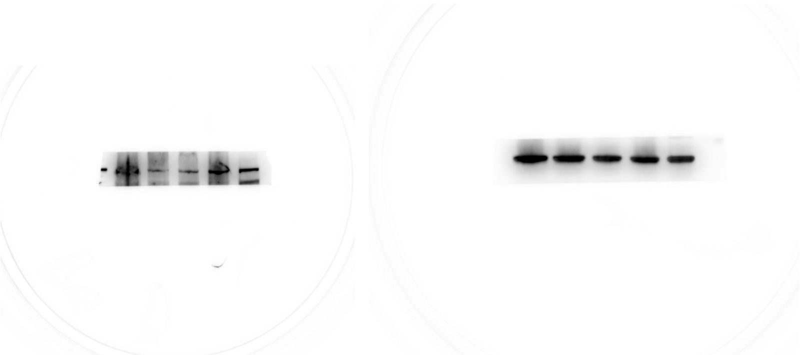

Fig 2A

KYSE450

GAPDH ROBO1

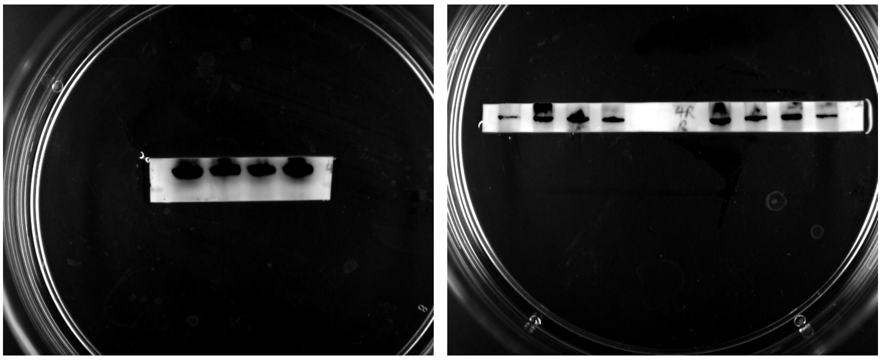

TE1

GAPDH ROBO1

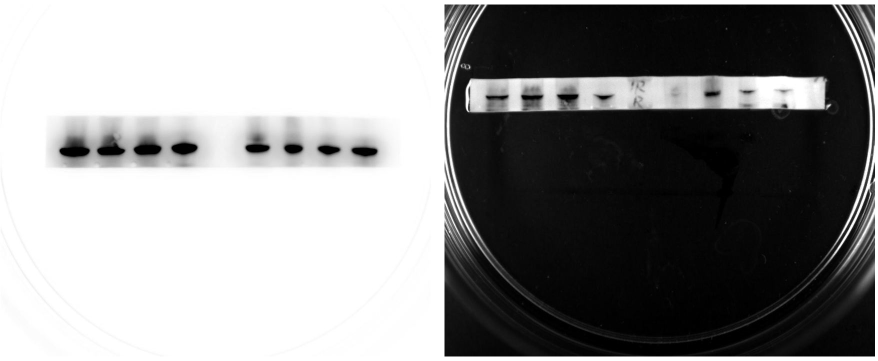

Fig 2B

KYSE450

GAPDH ROBO1

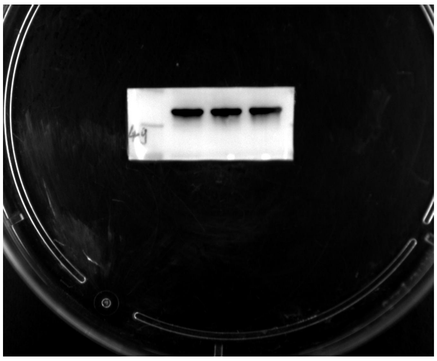

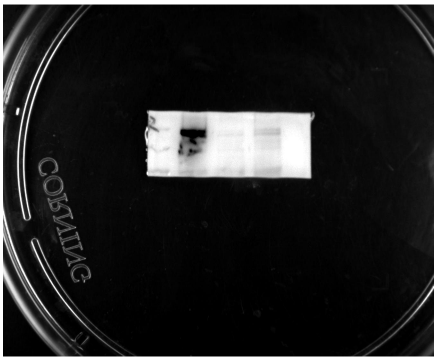

TE1

GAPDH ROBO1

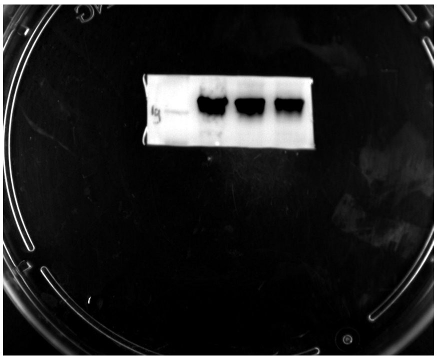

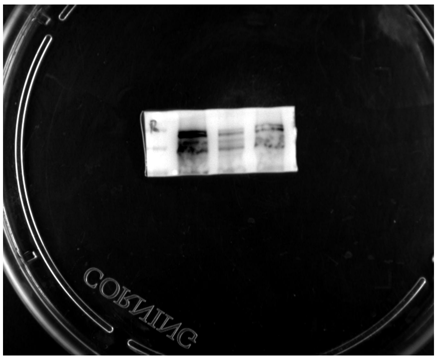

Fig 2G

KYSE450

GAPDH

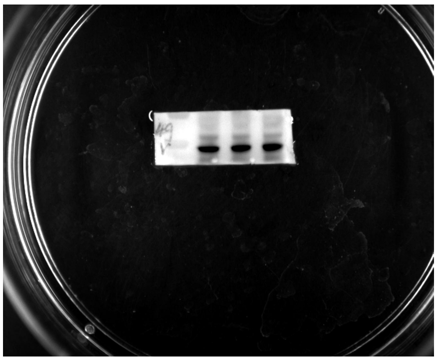

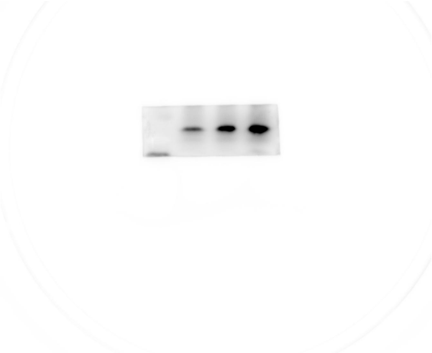

TE1

GAPDH H2AX

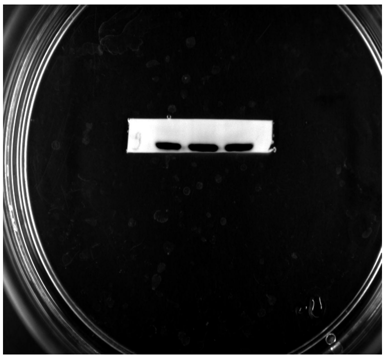

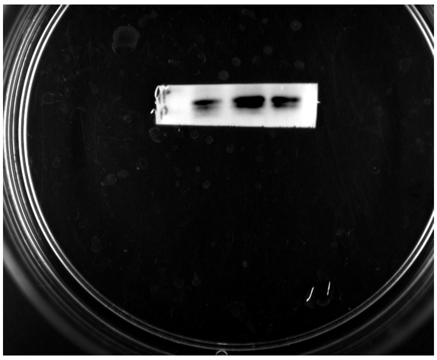

Fig 2I

TE1

GAPDH BCL2 ROBO1

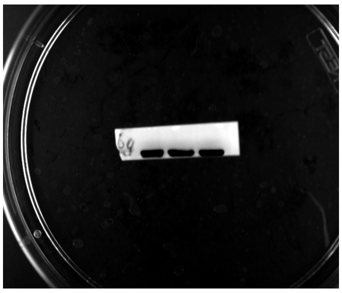
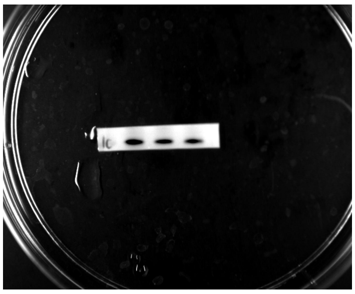
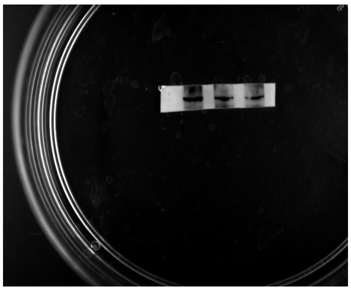

KYSE450

GAPDH BCL2

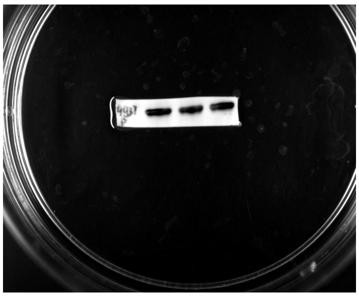
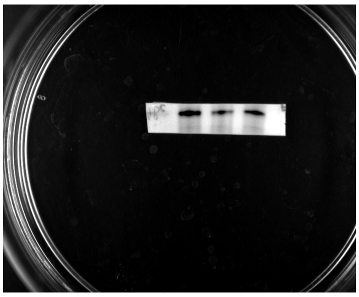
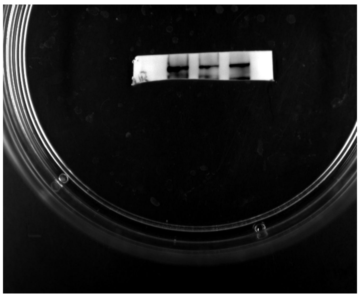

Fig 3A

KYSE450 TE1

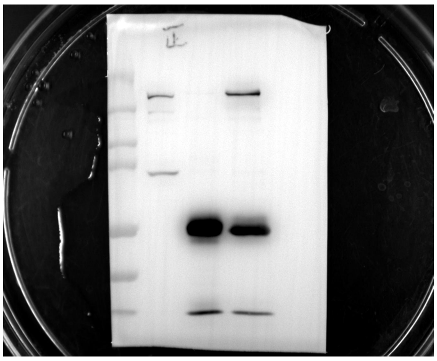
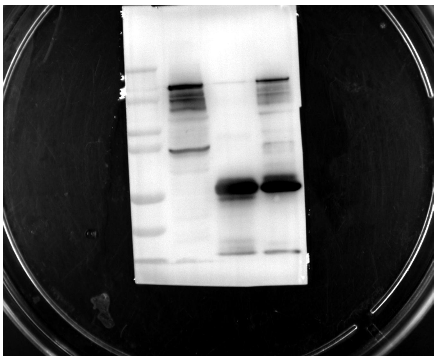

Fig 3E

TE1-GAPDH TE1-ROBO1 TE1-EIF3A

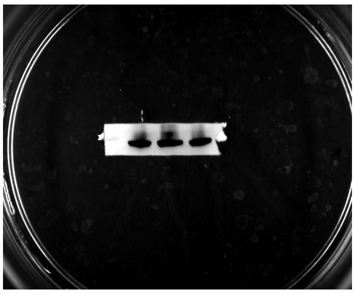
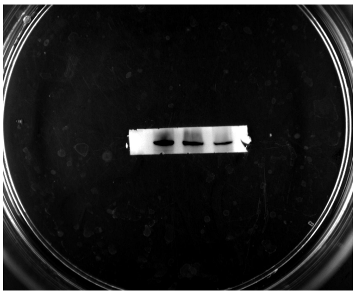
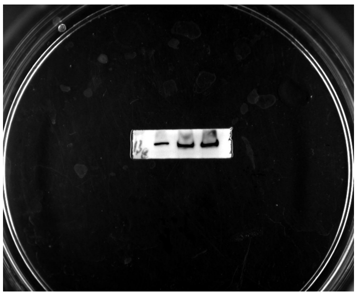

KYSE450-ROBO1 KYSE450-GAPDH KYSE450-EIF3A

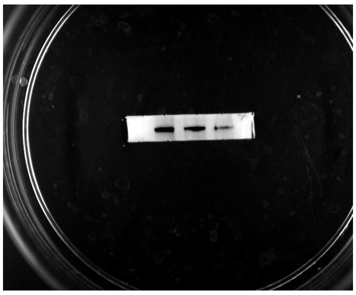
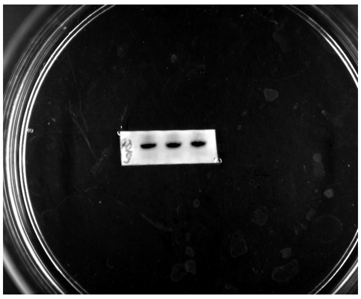
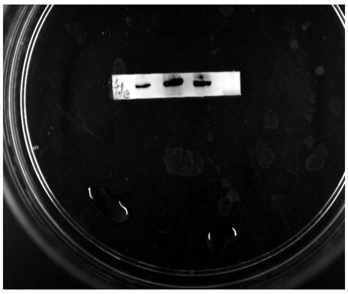

Fig 3C

KYSE450-EIF3A KYSE450-ROBO1

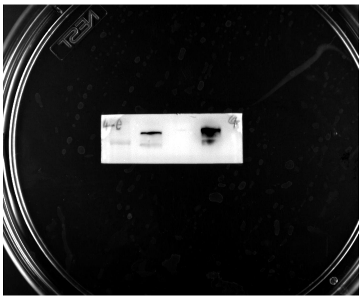
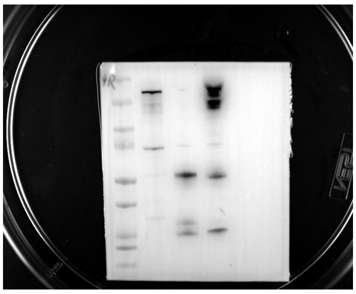

TE1-EIF3A

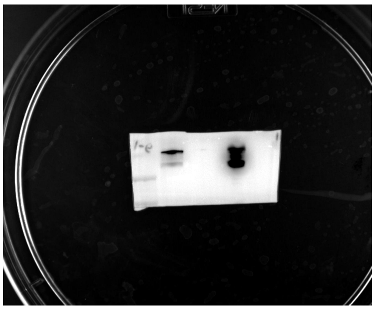
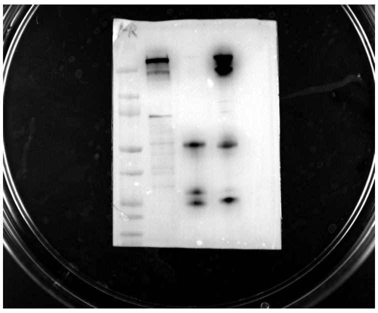

Fig 3G

KYSE450

Control shRNA

GAPDH EIF3A

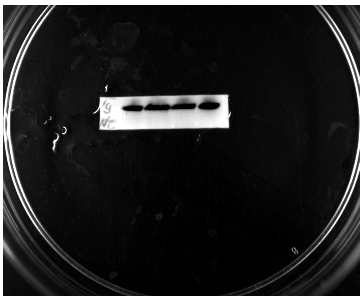
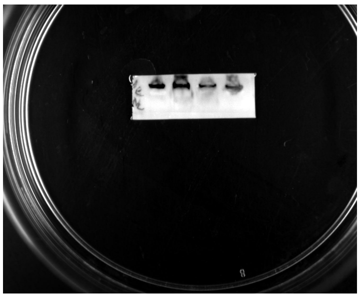

Robo1-shRNA1

GAPDH EIF3A

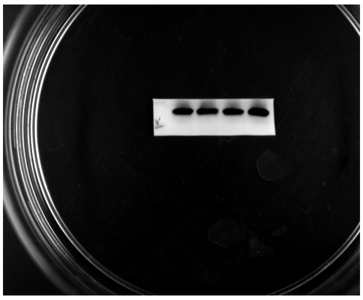
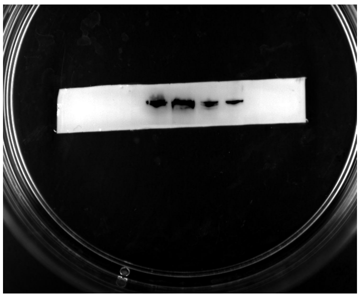

ROBO1-shRNA2

GAPDH EIF3A

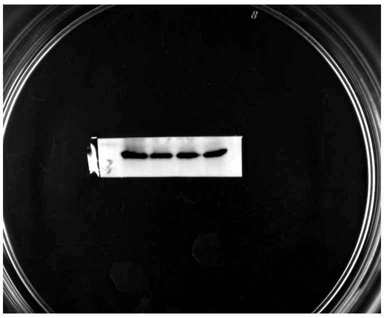
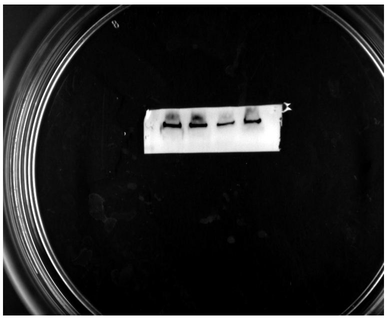

TE1-control shRNA

GAPDH EIF3A

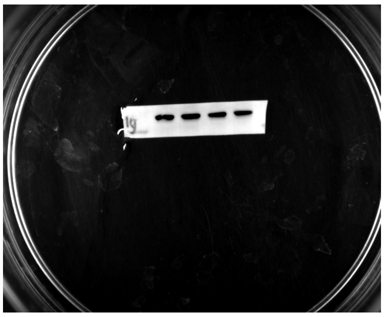
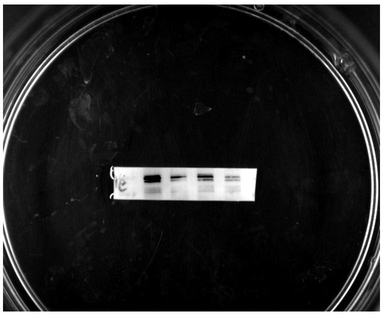

TE1-ROBO1 shRNA1

GAPDH EIF3A

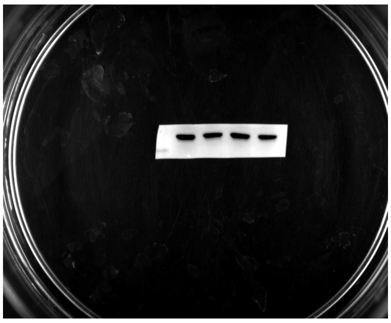
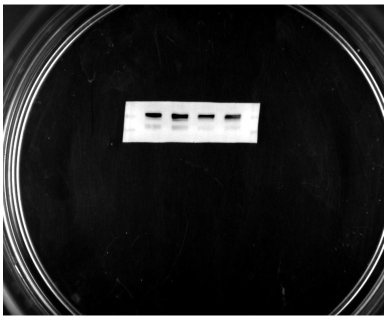

TE1-ROBO1 shRNA2

GAPDH EIF3A

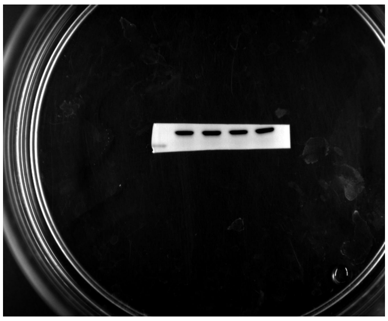 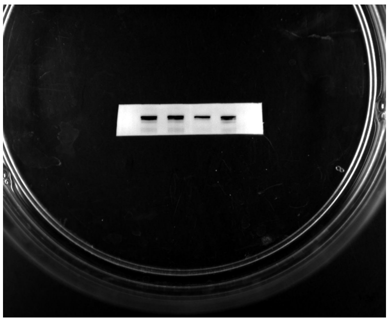

Fig 4A

KYSE450-ROBO1 KYSE450-GAPDH KYSE450-EIF3A

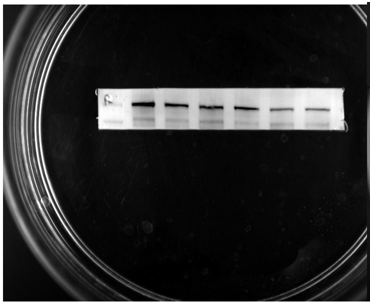 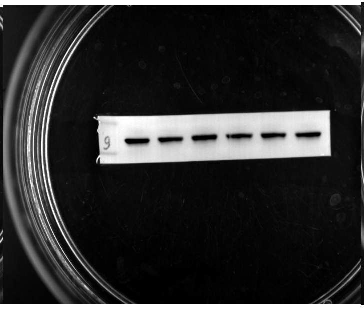 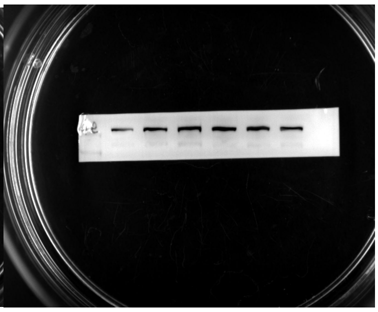

TE1-GAPDH TE1-ROBO1 TE1-EIF3A

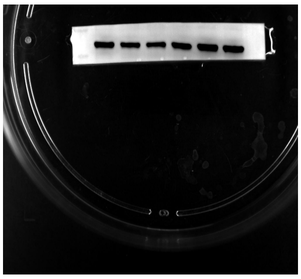 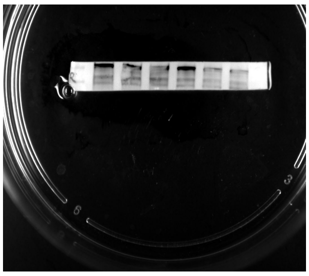 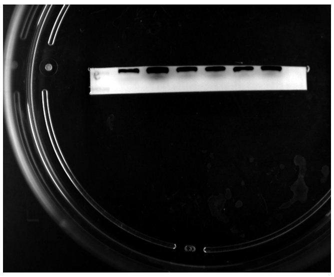

Fig 4B

TE1-EIF3A TE1-G3BP2 TE1-ROBO1

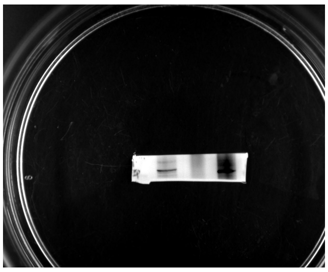 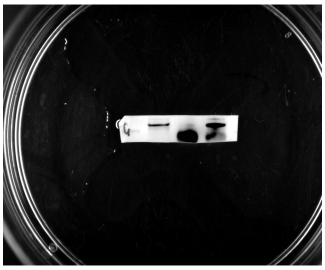 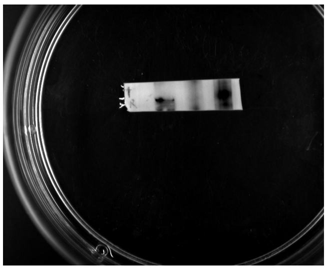

KYSE450-EIF3A KYSE450-G3BP2 KYSE450-ROBO1

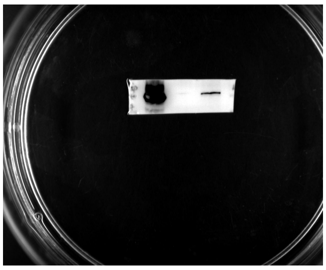

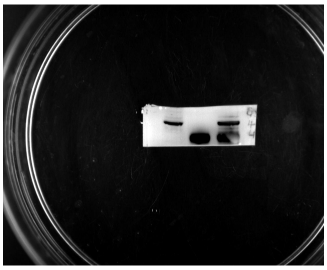

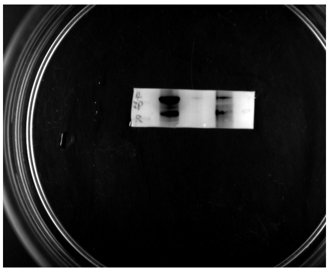

Fig 4D

KYSE450-GAPDH

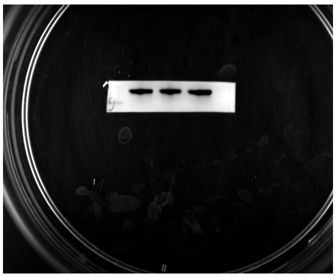

KYSE450-G3BP2

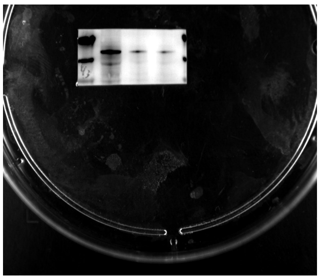

KYSE450-EIF3A

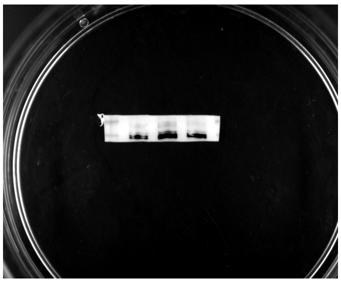

TE1-GAPDH

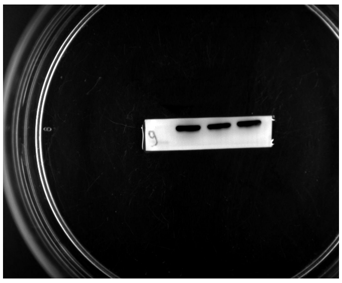

TE1-G3BP2

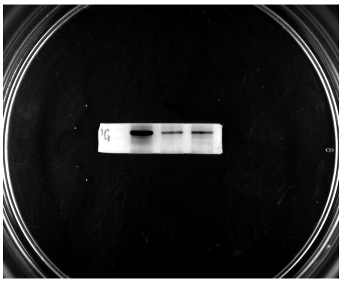

TE1-EIF3A

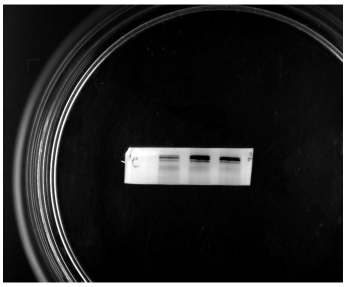

Fig 4F

CHX-GAPDH

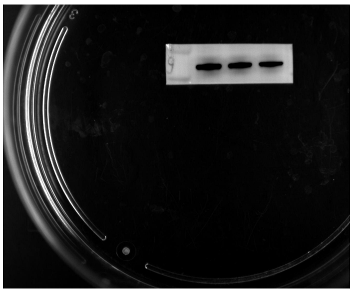

CHX-G3BP2

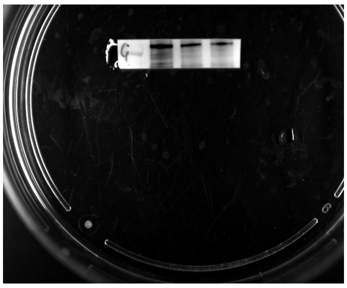

CHX-EIF3A

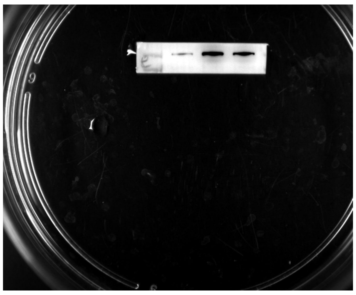

MG132-GAPDH

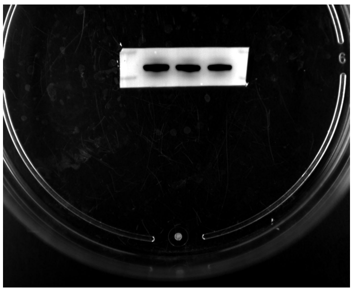

MG132-G3BP2

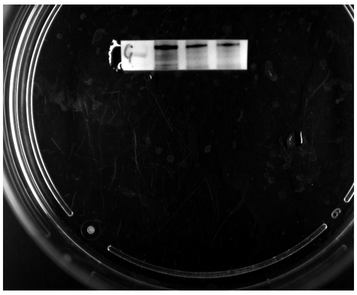

MG132-EIF3A

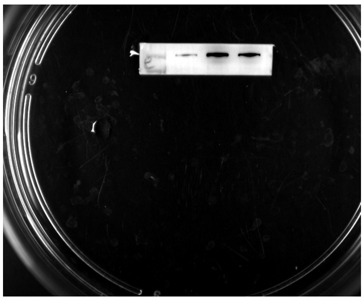

CQ-GAPDH CQ-G3BP2 CQ-EIF3A

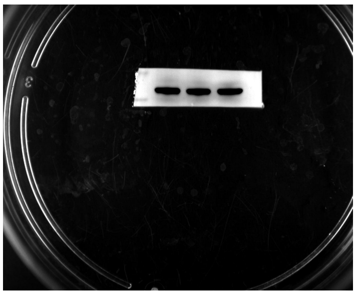
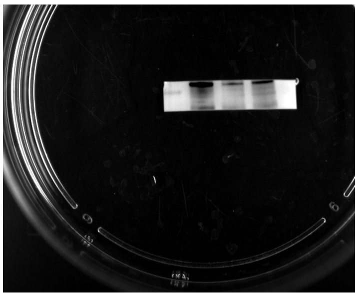
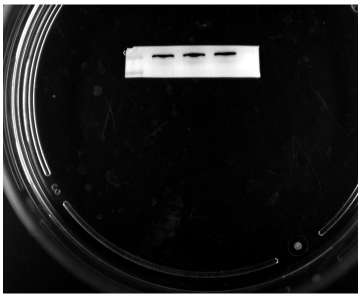

Fig 4G

KYSE450-LAMP1 KYSE450-LAMP2 KYSE450-TUBULIN

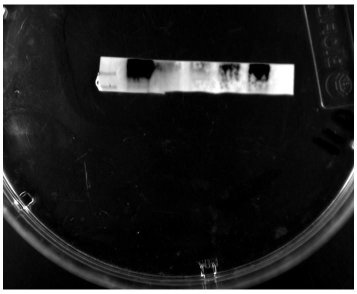
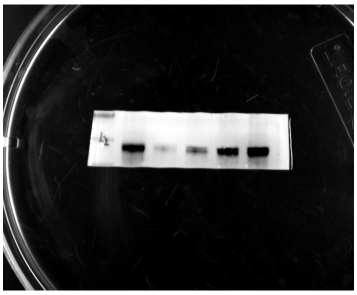
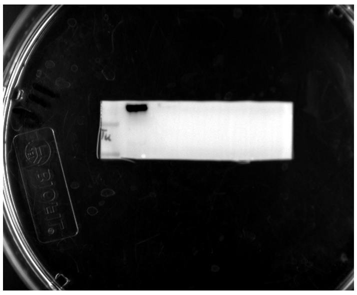

KYSE450-TOMM20 KYSE450-ROBO1 KYSE450-ERP72

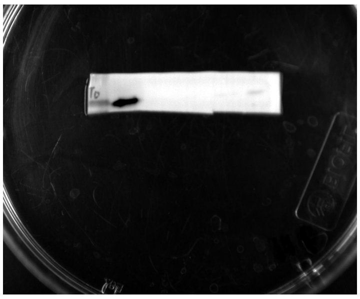
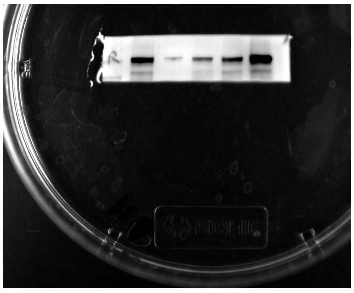
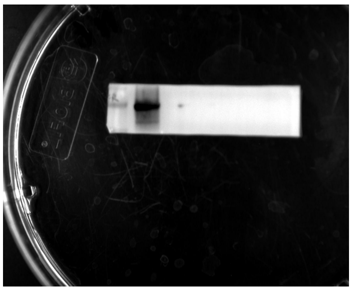

KYSE450-H3 KYSE450-G3BP2 KYSE450-EIF3A

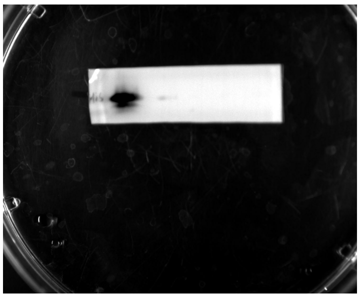
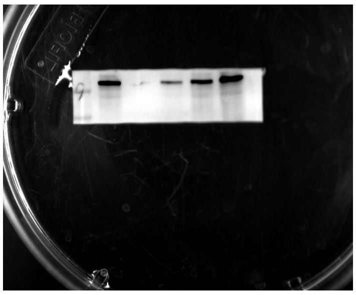
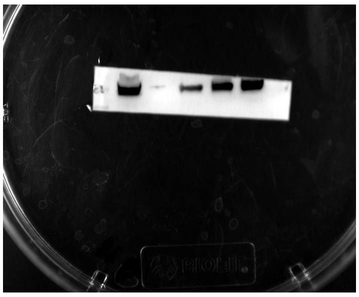

TE1-EIF3A TE1-G3BP2 TE1-LAMP2

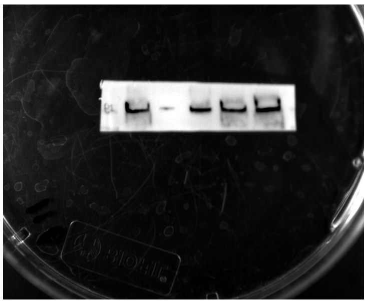
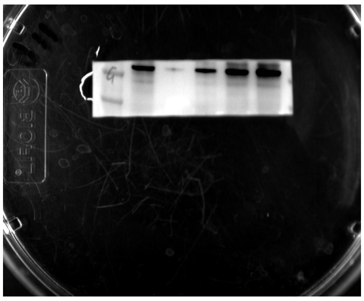
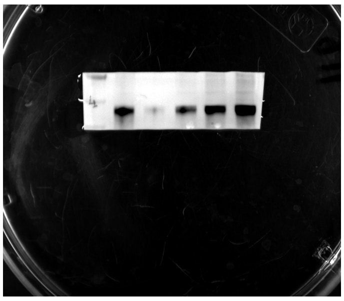

TE1-LAMP1 TE1-ERP72 TE1-HISTONE

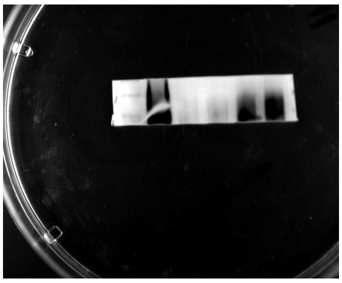 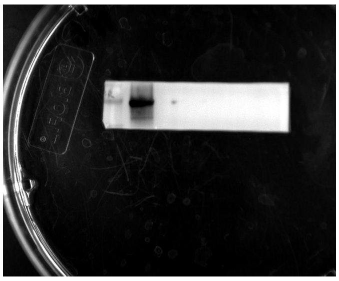 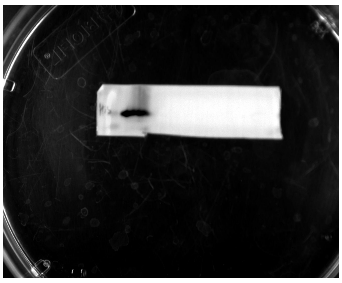

TE1-ROBO1 TE1-TOMM20 TE1-TUBULIN

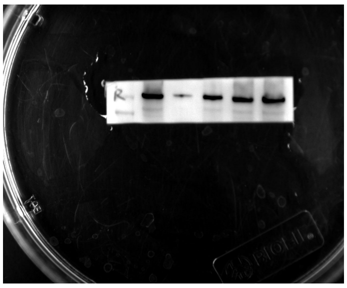 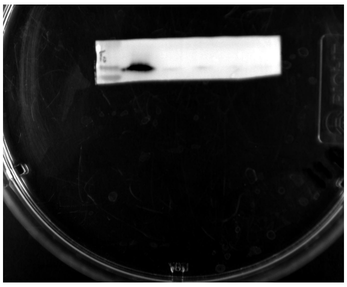 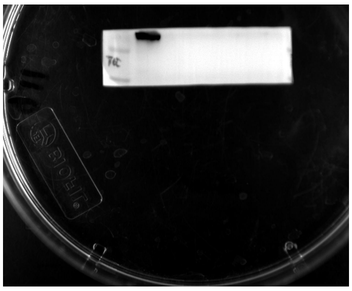

Fig 5E

TE1-H2AX TE1-G3BP2 TE1-GAPDH

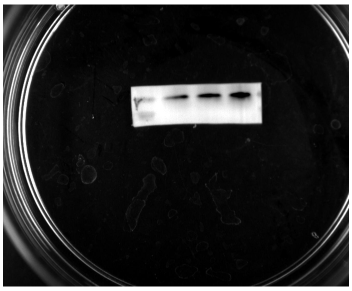 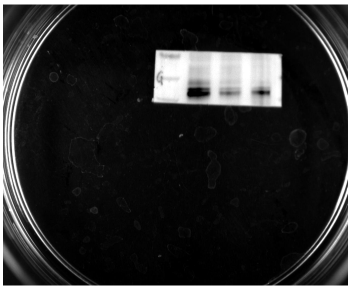 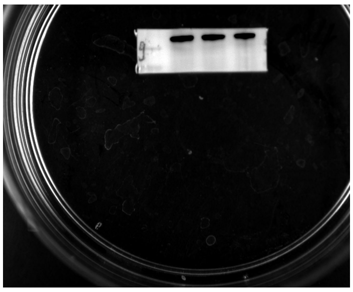

KYSE450-H2AX KYSE450-G3BP2

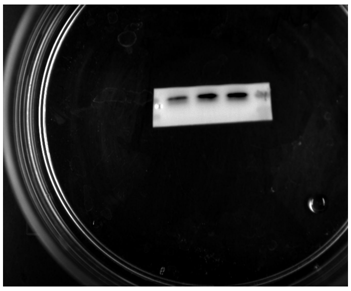 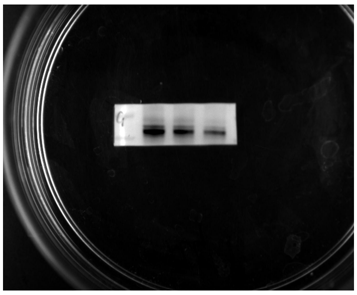 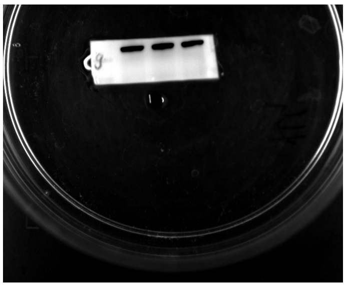

Fig 5H

TE1-G3BP2 TE1-GAPDH TE1-BCL2

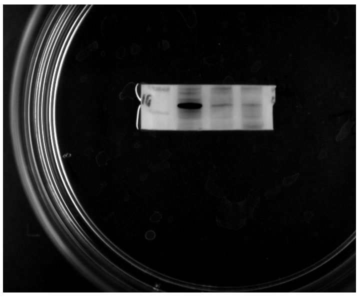 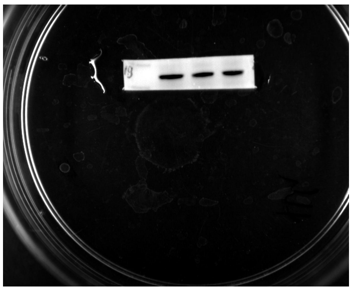 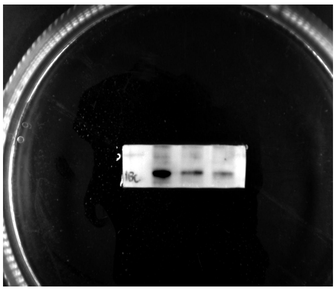

KYSE450-G3BP2 KYSE450-GAPDH KYSE450-BCL2

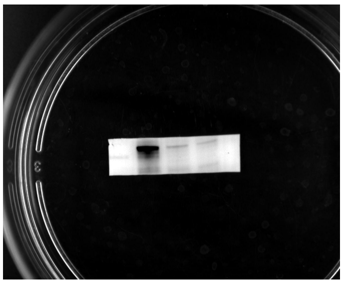

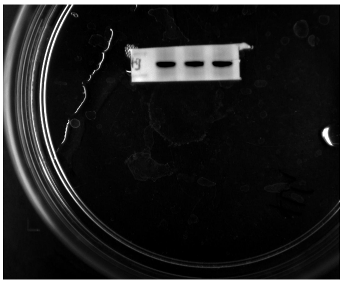

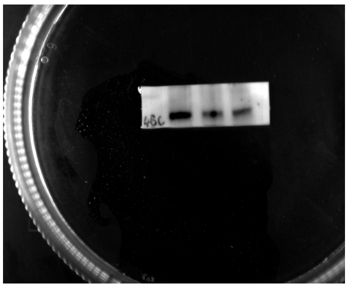

Fig 6B

KYSE450-GAPDH KYSE450-ROBO1 KYSE450-P53

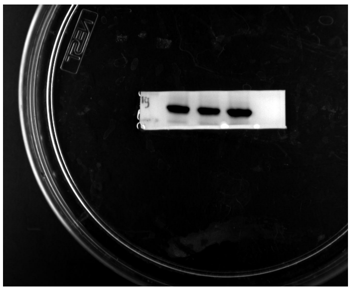

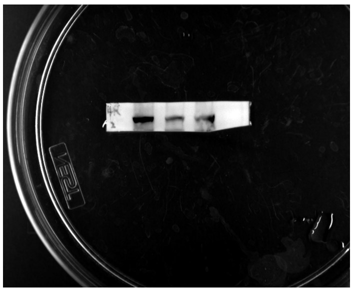

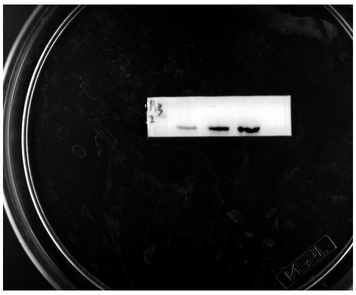

TE1-GAPDH TE1-ROBO1

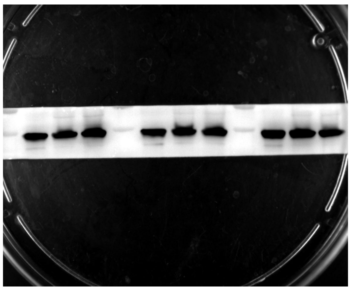

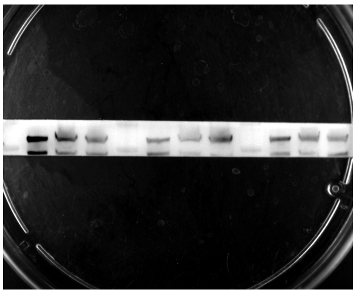

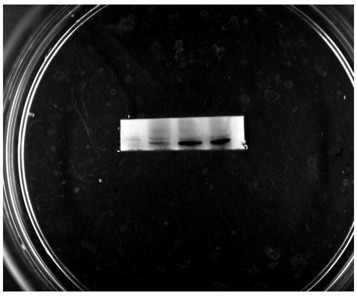

Fig 6D

KYSE450-CHX-GAPDH KYSE450-CHX-ROBO1 KYSE450-CHX-P53

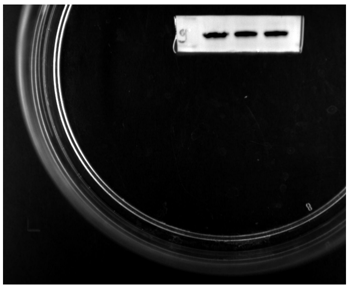

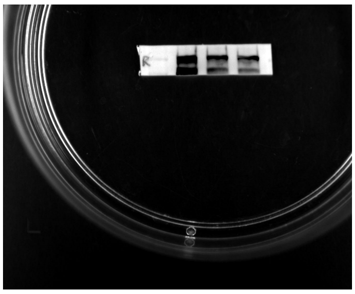

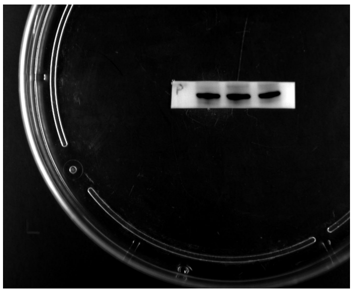

KYSE450-MG132-GAPDH KYSE450-MG132-ROBO1 KYSE450-MG132-P53

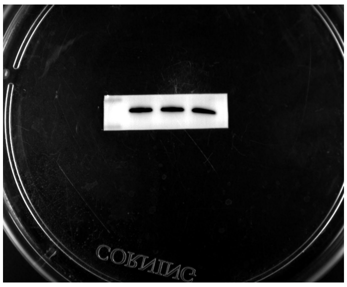 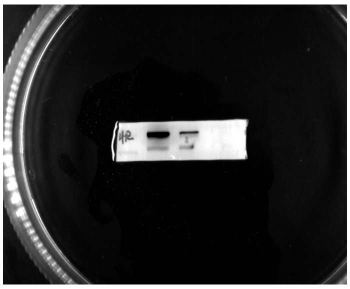 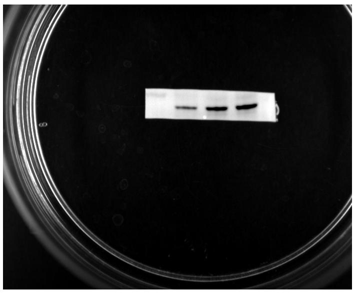

TE1-MG132-GAPDH TE1-MG132-ROBO1 TE1-MG132-P53

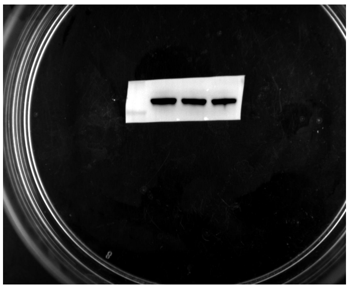 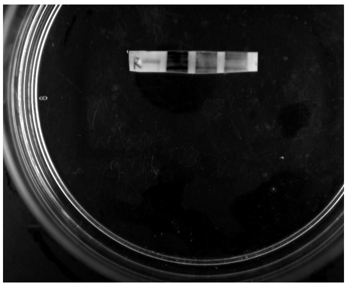 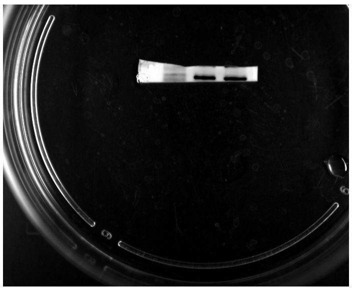

TE1-CHX-GAPDH TE1-CHX-ROBO1 TE1-CHX-P53

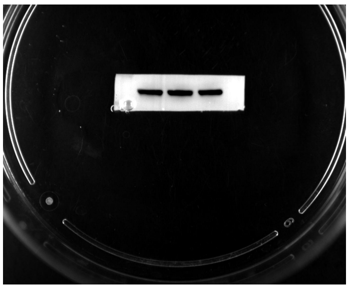 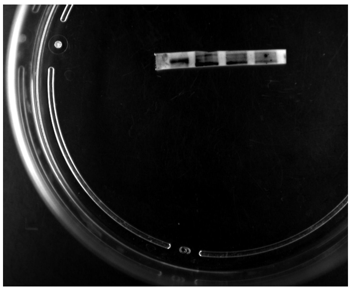 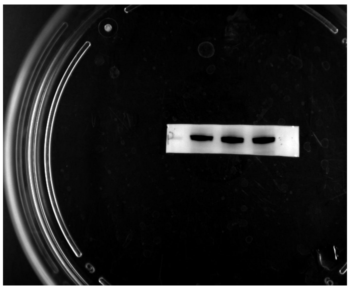

Fig 6E

GAPDH EIF3A

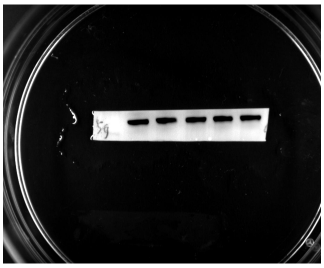 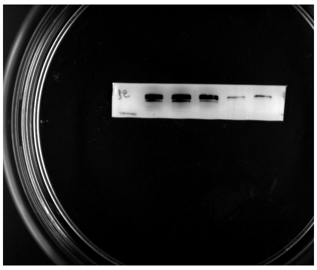

P53 ROBO1

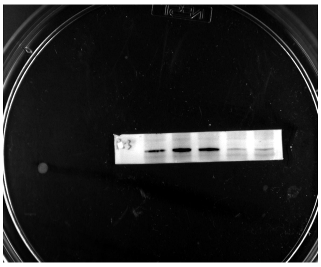 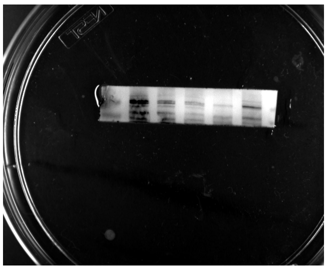

Fig 6F

GAPDH P53 EIF3A

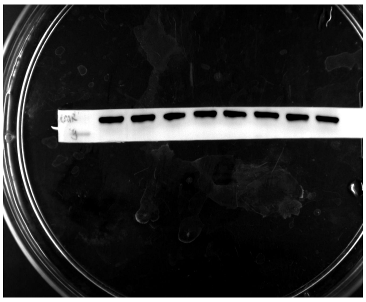
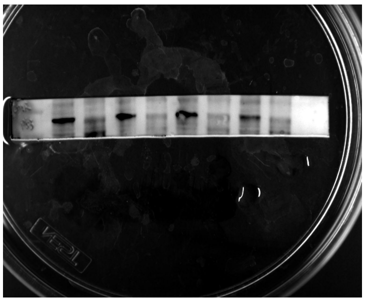
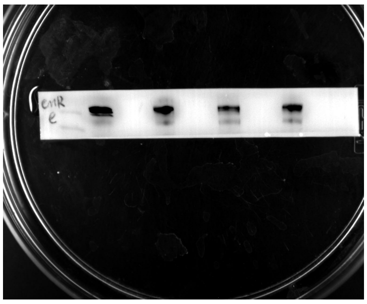

Fig 6G

KYSE450-GAPDH KYSE450-P53 KYSE450-XPA

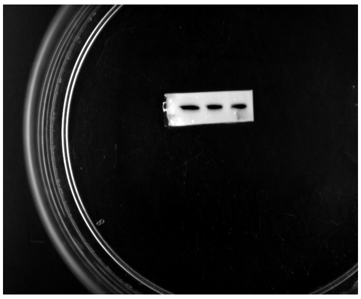
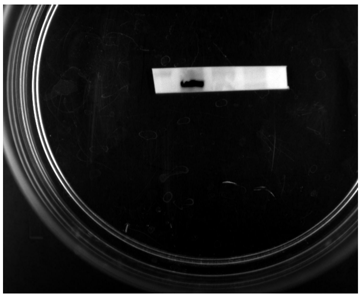
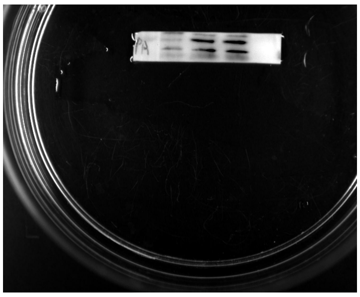

KYSE450-mTOR KYSE450-P-mTOR KYSE450-XPC

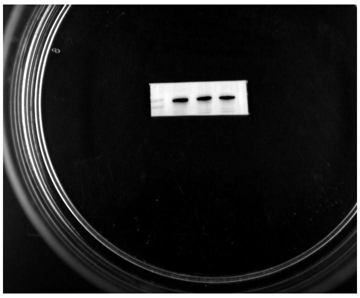
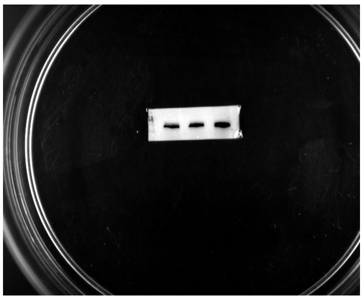
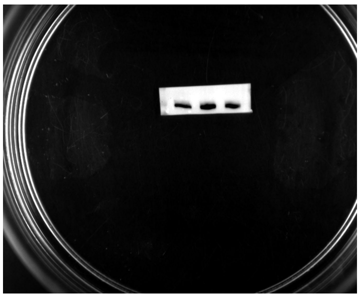

TE1-GAPDH TE1-P53 TE1-XPA

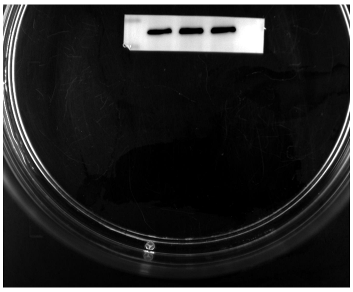
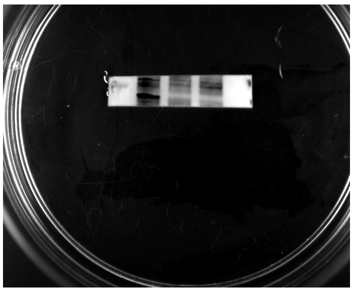
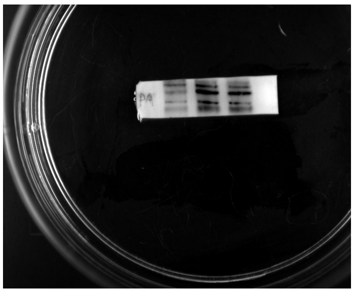

TE1-mTOR TE1-p-mTOR TE1-XPC

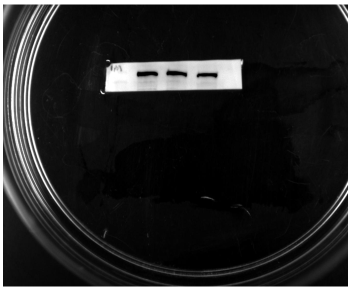
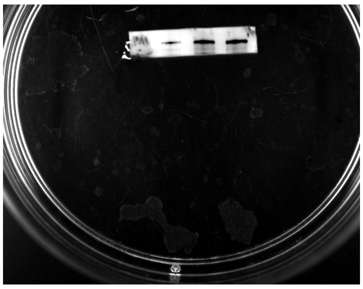
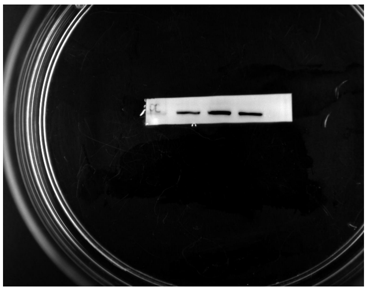

Fig 6H

KYSE450-GAPDH

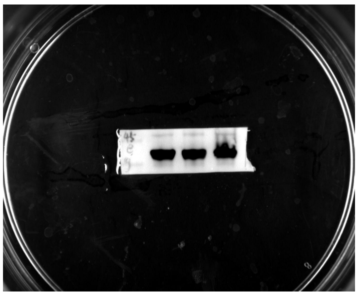

KYSE450-XPA

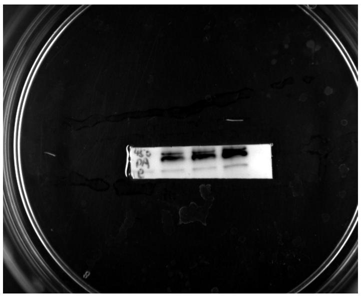

KYSE450-EIF3A

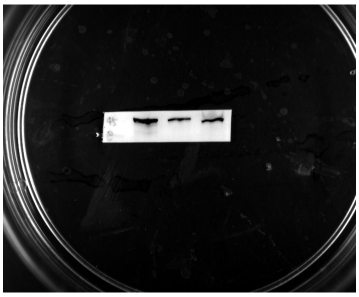

KYSE450-XPC

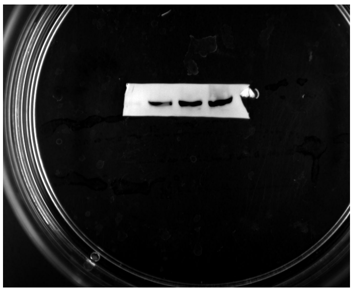

KYSE450-p-mTOR

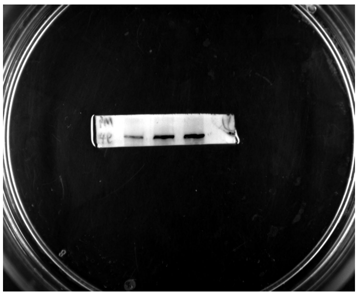

KYSE450-mTOR

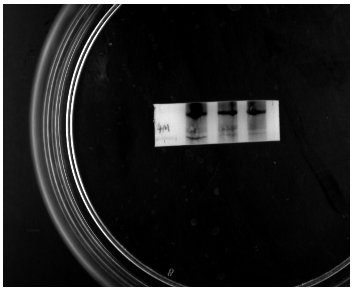

TE1-XPC

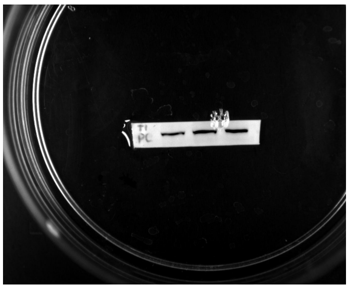

TE1-XPA

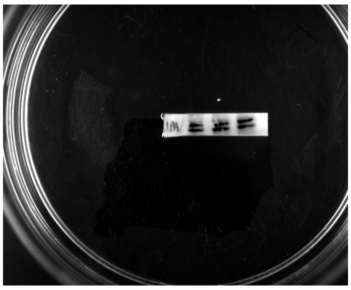

TE1-EIF3A

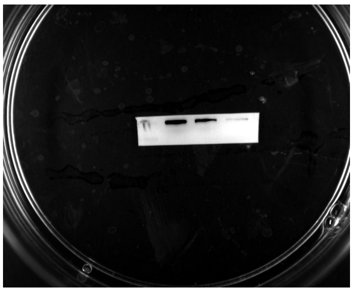

TE1-GAPDH

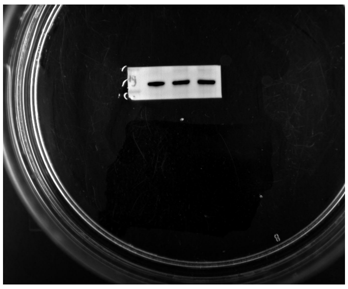

TE1-mTOR

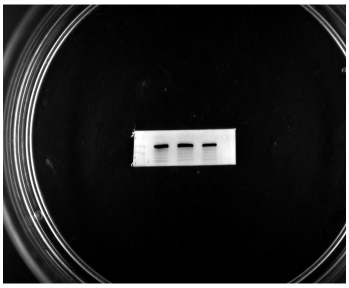

TE1-p-mTOR

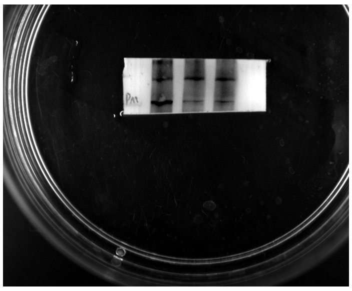

Fig 6I

KYSE450-GAPDH

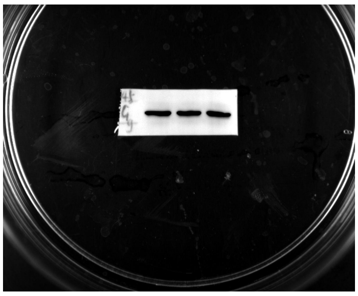

KYSE450-G3BP2

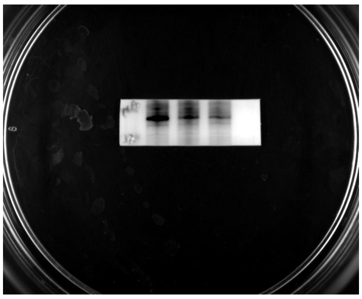

KYSE450-XPC

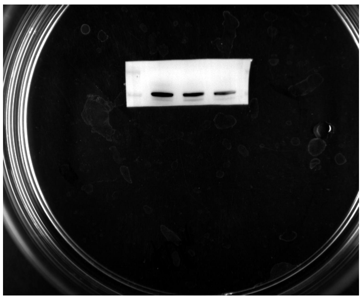

KYSE450-XPA KYSE450-mTOR KYSE450-p-mTOR

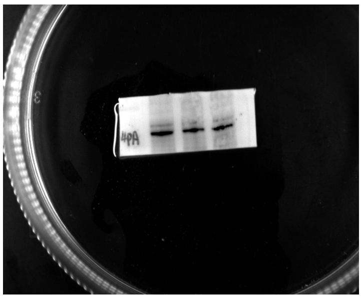 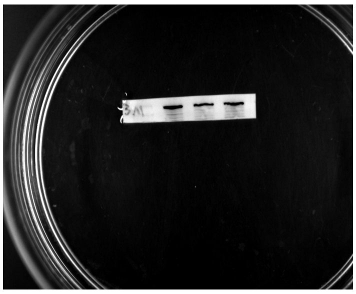 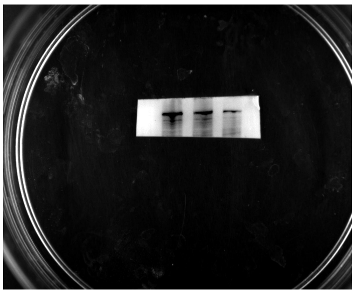

TE1-GAPDH TE1-G3BP2 TE1-XPC

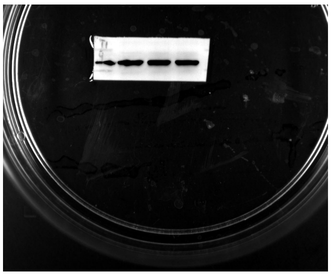 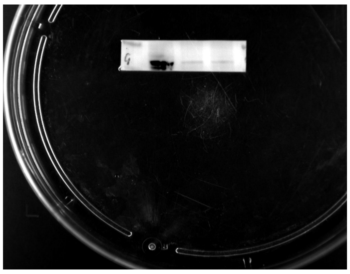 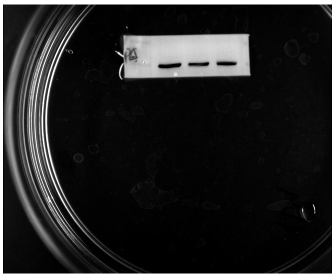

TE1-XPA TE1-mTOR TE1-p-mTOR

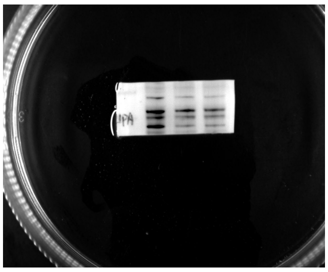 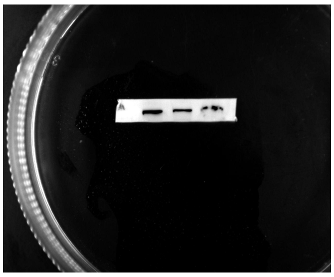 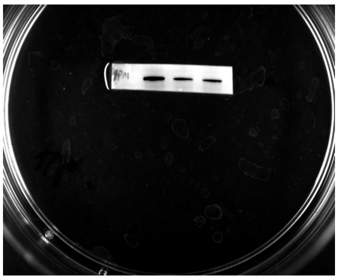

Fig 6J

KYSE450-GAPDH KYSE450-mTOR KYSE450-P-mTOR

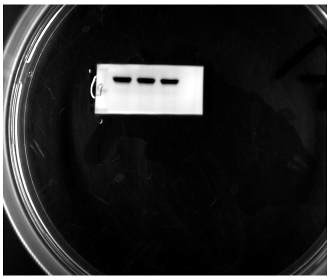 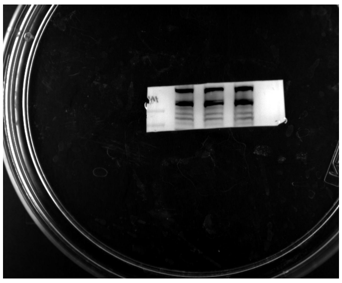 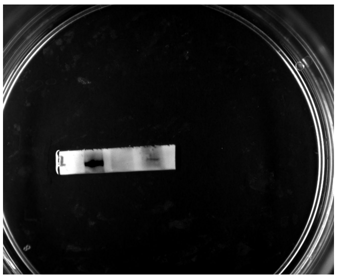

KYSE450-XPA KYSE450-XPC KYSE450-ROBO1

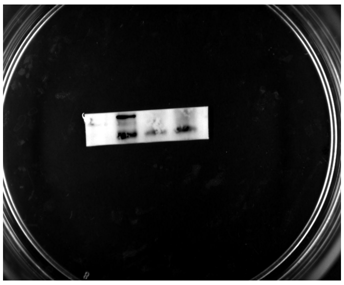

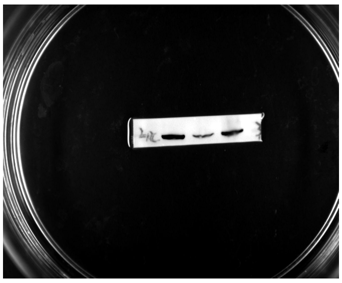

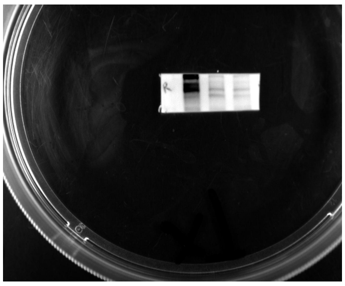

TE1-ROBO1 TE1-GAPDH TE1-XPC

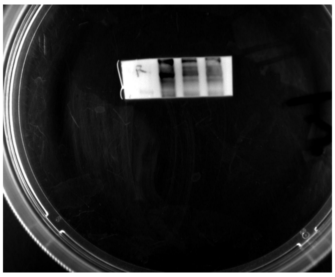

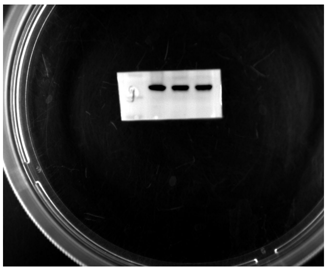

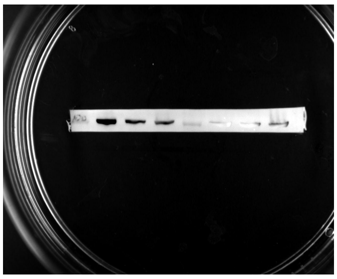

TE1-XPA TE1-p-mTOR TE1-mTOR

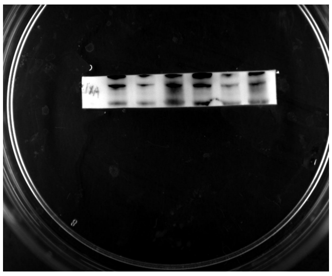

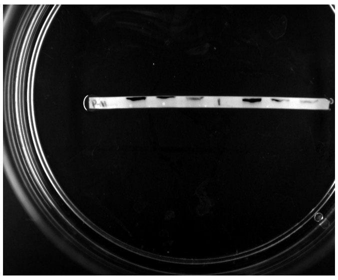

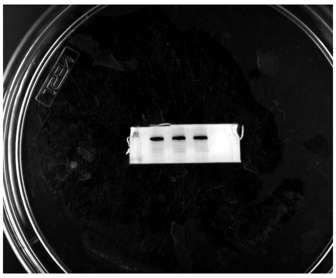

Supplement: Supplementary file 1 — Original Data [file 41419_2025_7604_MOESM1_ESM.pdf]
