# Supplementary material for: ROBO1 enhanced esophageal carcinoma cell radioresistance through accelerating G3BP2-mediated eIF3A degradation
Source: Cell Death Dis. 2025 Apr 6;16(1):256. doi: 10.1038/s41419-025-07604-1 (PMC11972380; doi:10.1038/s41419-025-07604-1)
Supplement: Supplementary file 2 — supplements [file 41419_2025_7604_MOESM2_ESM.doc]

Supplemental Figures


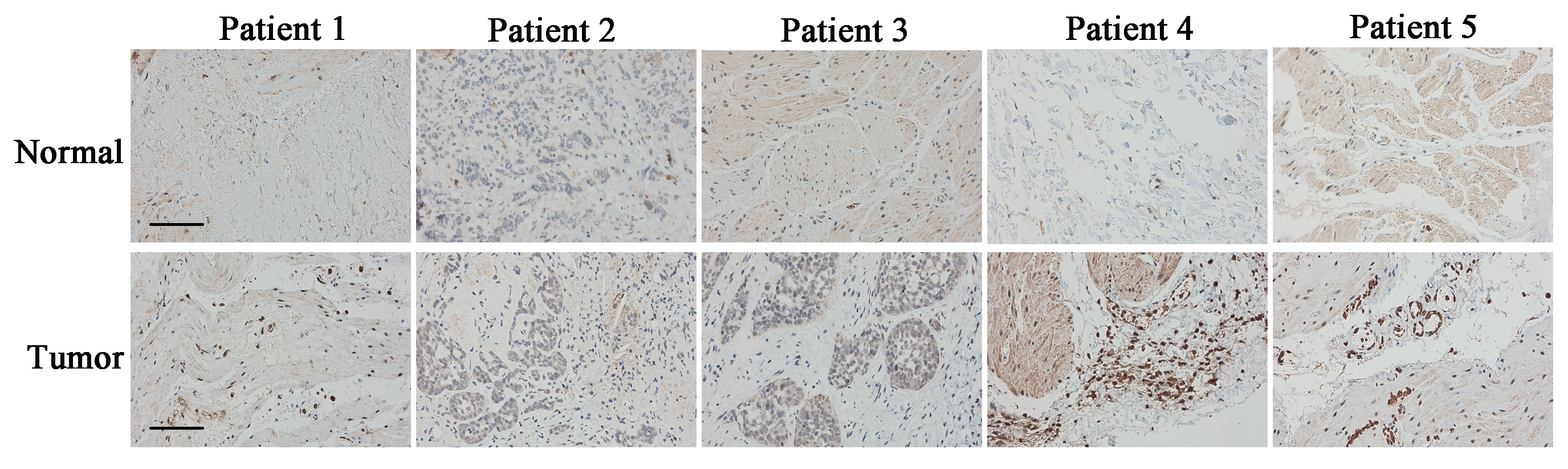


Figure S1. Immunohistochemical staining of ROBO1 in paired clinical ESCC cancerous tissues. Scale bars, 100μm.


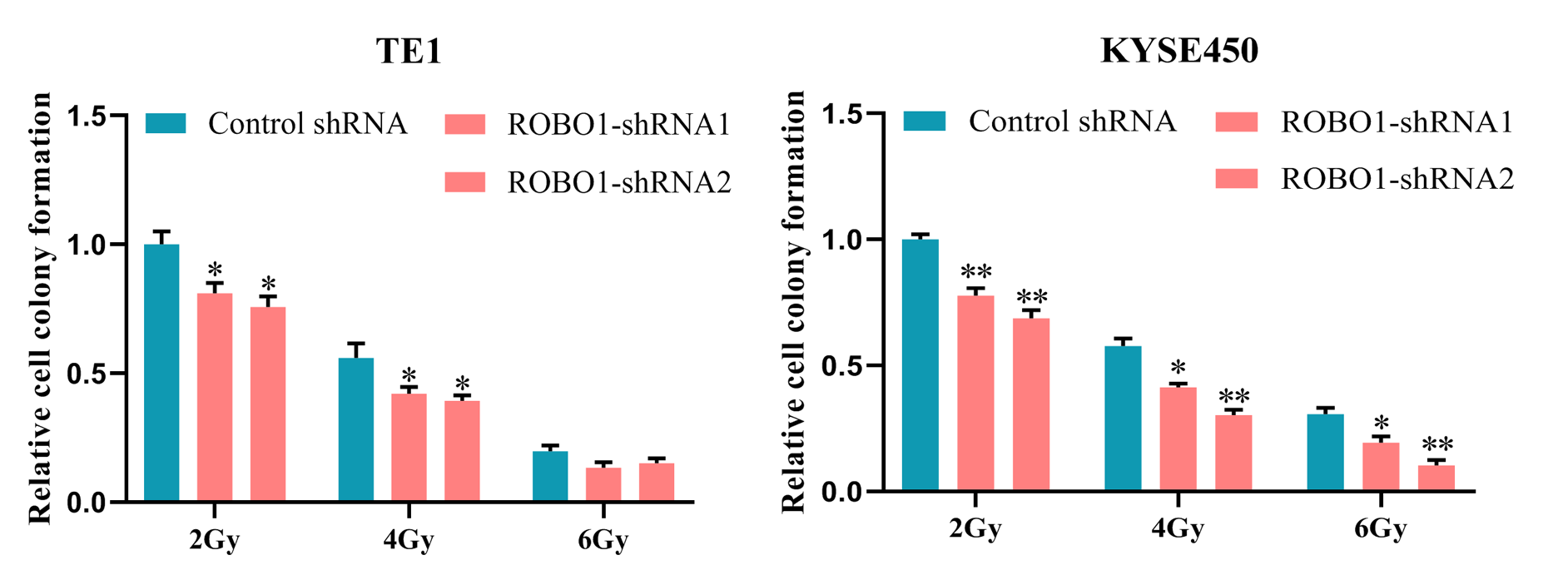


Figure S2. Cell colony formation quantification of Fig 2D. “*”, p<0.05; “**”, p<0.01.


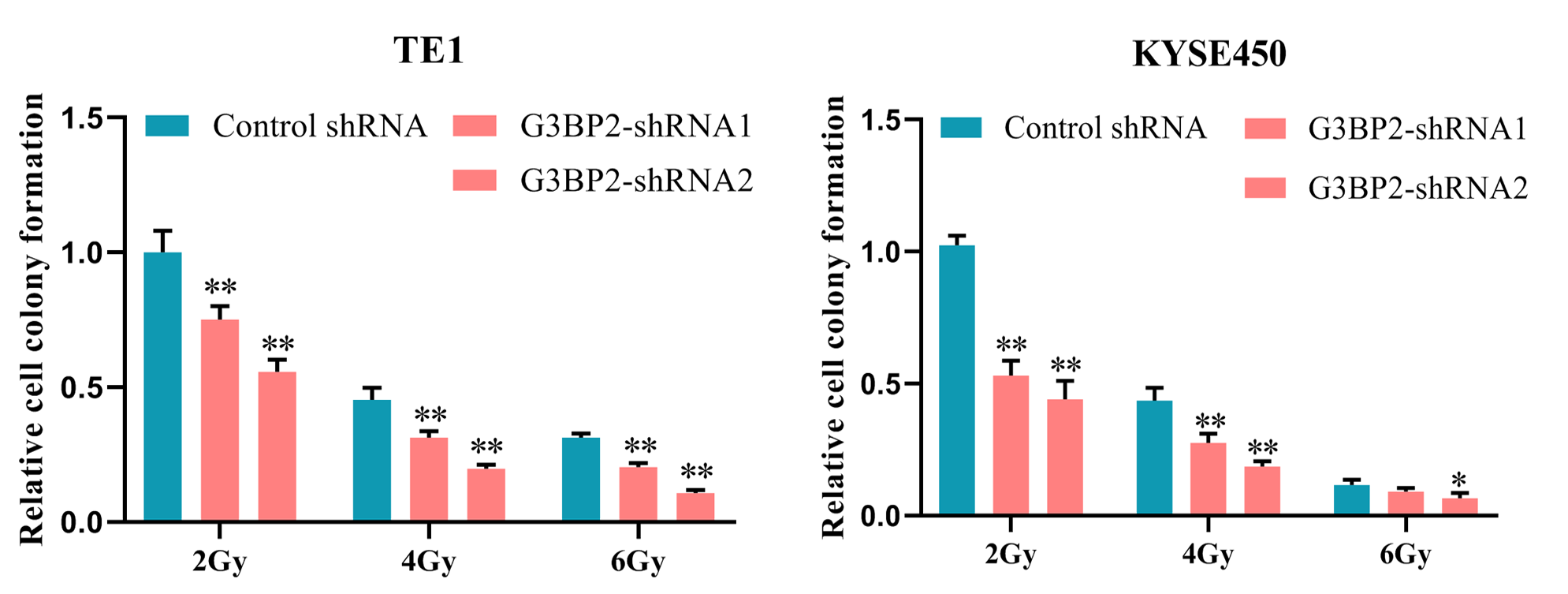


Figure S3. Cell colony formation quantification of Fig 5D. “*”, p<0.05; “**”, p<0.01.

Table 1. List of antibodies

| Antibodies | Brand | Cat.no |
| --- | --- | --- |
| ROBO1 | HUABIO, China | EM1901-72  ER1706-24 |
| GAPDH | Bioss, China | bsm-52262R |
| Ki67 | Servicebio, China | GB121141-50 |
| γ-H2A.X | Proteintech, China | 29380-1-AP |
| BCL2 | Proteintech, China | 12789-1-AP |
| eIF3A | Cell Signaling Technology  Proteintech, China | 3411T  67713-1-Ig |
| G3BP2 | Proteintech, China | 16276-1-AP  68580-1-Ig |
| LAMP2 | Servicebio, China  Abcam, USA | GB11848-100  ab199946 |
| LAMP1 | Cell Signaling Technology  Servicebio, China | 15665T  GB112949-100 |
| P53 | Cell Signaling Technology | 9282T |
| p-mTOR | Cell Signaling Technology | 5536T |
| mTOR | Cell Signaling Technology | 2983T |
| XPA | Immunoway, USA | YT4914 |
| XPC | Immunoway, USA | YT6473 |
| Tubulin | Proteintech, China | 14555-1-AP |
| TOM20 | Beyotime, China | AF1717 |
| Histone H3 | Beyotime, China | AF0009 |
| ERp72 | Proteintech, China | 14712-1-AP |

Table 2. Primers used for qPCR

| Gene | Forward (5’-3’) | Reverse (5’-3’) |
| --- | --- | --- |
| ROBO1 | ACACCCGTAAAAGTGACGCT | TAGGTACAGGGTCACCTCGG |
| G3BP2 | AATCTCAGCCACCTCGTGTG | TGCCTGGTCTTGGTCCTCTA |
| P53 | AGTCACAGCACATGACGGAG | GCCAGACCATCGCTATCTGA |
| GAPDH | GCACCGTCAAGGCTGAGAAC | TGGTGAAGACGCCAGTGGA |
| eIF3A | CAACTGGAACGGGCCATAGT | ACTCAGGGTCCGAGAAGTGT |

Table 3. Sequences for RNA interference

| GENE | Sequence(5’-3’) |
| --- | --- |
| P53-siRNA1 | CCCGGACGAUAUUGAACAATT |
| P53-siRNA2 | CCAUCUACAAGCAGUCACATT |
| G3BP2-siRNA1 | GGGAGUUUGUGAGGCAAUATT |
| G3BP2-siRNA2 | GCCACAUGAUAUUGAUGAATT |
| ROBO1-shRNA1 | GGAAAGCTCATGATCACTTAC |
| ROBO1-shRNA2 | GAGGGCAGCTAATGCATAT |
| G3BP2-shRNA1 | GGGAGUUUGUGAGGCAAUATT |
| G3BP2-shRNA2 | GCCACAUGAUAUUGAUGAATT |
| eIF3A-siRNA1 | GCGCCAACGAAUUUCUUGATT |
| eIF3A-siRNA2 | GGCUCUUGAACAUAAGAAUTT |
